# A High Epigenetic Risk Score Shapes the Non-Inflamed Tumor Microenvironment in Breast Cancer

**DOI:** 10.3389/fmolb.2021.675198

**Published:** 2021-07-26

**Authors:** Dong Zhang, Yingnan Wang, Qifeng Yang

**Affiliations:** ^1^ Department of Breast Surgery, Qilu Hospital, Cheeloo College of Medicine, Shandong University, Jinan, China; ^2^ Department of Clinical Medicine, Cheeloo College of Medicine, Shandong University, Jinan, China; ^3^ Department of Pathology Tissue Bank, Qilu Hospital, Shandong University, Jinan, China

**Keywords:** breast cancer, prognosis, DNA methylation, tumor microenvironment, therapeutic target

## Abstract

**Background:** Epigenetic dysregulation via aberrant DNA methylation has gradually become recognized as an efficacious signature for predicting tumor prognosis and response to therapeutic targets. However, reliable DNA methylation biomarkers describing tumorigenesis remain to be comprehensively explored regarding their prognostic and therapeutic potential in breast cancer (BC).

**Methods:** Whole-genome methylation datasets integrated from the Cancer Genome Atlas (TCGA) and Gene Expression Omnibus (GEO) database were profiled (*n* = 1,268). A three-stage selection procedure (discovery, training, and external validation) was utilized to screen out the prominent biomarkers and establish a robust risk score from more than 300,000 CpG sites after quality control, rigorous filtering, and reducing dimension. Moreover, gene set enrichment analyses guided us to systematically correlate this epigenetic risk score with immunological characteristics, including immunomodulators, anti-cancer immunity cycle, immune checkpoints, tumor-infiltrating immune cells and a series of signatures upon modulating components within BC tumor microenvironment (TME). Multi-omics data analyses were performed to decipher specific genomic alterations in low- and high-risk patients. Additionally, we also analyzed the role of risk score in predicting response to several treatment options.

**Results:** A 10-CpG-based prognostic signature which could significantly and independently categorize BC patients into distinct prognoses was established and sufficiently validated. And we hypothesize that this signature designs a non-inflamed TME in BC based on the evidence that the derived risk score is negatively correlated with tumor-associated infiltrating immune cells, anti-cancer immunity cycle, immune checkpoints, immune cytolytic activity, T cell inflamed score, immunophenoscore, and the vast majority of immunomodulators. The identified high-risk patients were characterized by upregulation of immune inhibited oncogenic pathways, higher TP53 mutation and copy number burden, but lower response to cancer immunotherapy and chemotherapy.

**Conclusion:** Our work highlights the complementary roles of 10*-*CpG-based signature in estimating overall survival in BC patients, shedding new light on investigating failed events concerning immunotherapy at present.

## Introduction

Breast cancer (BC) ranks third among the most common malignancies and is the leading cause of cancer-related death in females (Lin et al., 2019; Li et al., 2019b). Currently, the improvement of mammographic screening has made great progress in early-stage diagnosis of BC. Moreover, there are a series of systematic treatments for BC, including surgical resection, chemotherapy, radiotherapy, endocrine therapy, and alternative molecule-targeted therapy (e.g., trastuzumab and pertuzumab) (Akram et al., 2017). However, postoperative local or distant recurrence rate remains high, even for patients who have received conventional therapies in the early stage, causing a pessimistic mortality rate within BC patients at present (Spronk et al., 2018; Chen et al., 2019). This could be attributed to the restricted and incomprehensive understanding of BC heterogeneity concerning carcinogenesis, invasiveness, progression, and metastasis (Tazaki et al., 2013). Molecular characteristics that are reliably related to BC prognosis and patient survival would have tremendous value in guiding clinical management of BC. Hence, a deeper understanding of BC functional pathways, as well as the development of novel crucial biomarkers with biological background, for early diagnosis and prognostic prediction in BC patients, is urgently needed.

Although previous studies at different omics levels, such as somatic mutations, gene expression, non-coding RNA, and copy number variations, have revealed numerous promising biomarkers relevant to BC carcinogenesis (Zhang et al., 2018a; Fan et al., 2019; He et al., 2019; Li et al., 2019a), the contribution of epigenetic alterations including DNA methylation in human disease, particularly in cancer, has also been widely recognized (Khaled and Bidet, 2019; Luo et al., 2020; Yang et al., 2020). From the point of view of mechanism, DNA methylation, as inherently reversible changes to repress the transcriptional activities and interact with various negative and positive feedback processes, plays decisive roles in both physiological and pathological regulation of cellular fate (Drake and Søreide, 2019). Accumulated evidence highlights the multifaceted DNA methylation of 5′-cytosine-phosphate-guanine-3′ (CpG) sites in various cancer hallmarks, including modulating energy metabolism and angiogenesis, sustaining proliferation signaling, epithelial-mesenchymal transition, invasiveness, and metastasis, mainly via promoting the activation of oncogenes and silencing of tumor suppressor genes (Pasculli et al., 2018; Drake and Søreide, 2019). The tumor microenvironment, intra-tumoral cell typing, and subsequent response to immunotherapy could also be evaluated and characterized by DNA methylation profiling (Jeschke et al., 2015; Jeschke et al., 2017; Calle-Fabregat et al., 2020). More importantly, DNA methylation offers feasible clues to assist the early detection and prognosis of different cancers, using the early onset of global alterations within DNA methylation profiling in cancer initiation and significantly distinct methylation patterns between tumor and normal tissues (Szyf, 2012).

Recent advances of high-throughput technology, combined with the availability of public, large-scale sequencing datasets, have opened a new area for defining the genome-wide landscape of BC and provided opportunities but daunting challenges to identify potentially reliable BC biomarkers (Jiang et al., 2019; Xuchao et al., 2019). Preliminary investigation of profiling arrays has proposed several methylation-based signatures for survival stratification in patients with BC (Du et al., 2019; Tao et al., 2019). Unfortunately, none has yet been incorporated in clinical practice owing to issues such as model overfitting on a single cohort and insufficient external validation. Moreover, the aforementioned analyses have not elucidated the complicated regulatory network that governed BC biological processes, and clinical characteristics were not included in the predictive models for further assessment.

In the current study, we identified CpG sites that are specifically and significantly correlated with BC prognosis and then developed a multi-CpG classifier by mining DNA methylation profiles from the Cancer Genome Atlas (TCGA) project. With sufficient validation of two independent sample sets, we proved the stability and reliability of our established model. In addition, pivotal biological processes underlying the prognostic signature were revealed via a series of bioinformatics analyses, and the correlations of CpG -based signature with tumor microenvironment (TME) were also comprehensively evaluated. To further leverage the complementary value of molecular and clinical characteristics, we integrated the CpG-based signature and clinicopathological risk factors to build a composite nomogram, which improved the risk stratification of BC patients.

## Materials and Methods

### Data Acquisition and Pre-Processing

Level 3 DNA methylation profiles using the Illumina Infinium HumanMethylation450 BeadChips Assay, including 785 BC patients, were downloaded and integrated from TCGA website (https://portal.gdc.cancer.gov) via “TCGA-Assembler 2” R package (https://github.com/compgenome365/TCGA-Assembler-2) (Tomczak et al., 2015; Wei et al., 2018), and the genomic manifest of each CpG site was annotated using “IlluminaHumanMethylation450kanno.ilmn12.hg19” R package (version 0.6.0) (Aryee et al., 2010). For each CpG locus, the methylation level was represented by the β value (β = M/(M+U)), which ranges from 0 to 1, corresponding to unmethylated and fully methylated. Low quality probes were excluded if they met the following criteria: 1) failed detection in more than 25% samples; 2) not uniquely mapped to the human reference genome; 3) located on unstable genomic sites, including single-nucleotide polymorphisms (SNP) sites and Y chromosome; or 4) missed annotations to parent genes. Furthermore, the remaining CpG sites with unavailable methylation levels (NAs) were imputed based on k-nearest neighbors (KNN) algorithm via “impute” R package (version 1.56.0).

Level 4 RNA sequencing data (FPKM normalized) based on the Illumina HiSeq RNA-seq platform of BC samples were obtained from GDC data portal of TCGA website via “TCGAbiolinks” R package (version 2.10.5) (Colaprico et al., 2016). The ensemble IDs were annotated to gene symbols using “gencode.v22.annotation.gtf” file (www.gencodegenes.org/human/releases.html) (Paik and Hancock, 2012). And the FPKM values were further transformed into transcripts per kilobase million (TPM) values, which are more reliable and recommended for inter-group comparisons of expressive abundance (Wagner et al., 2012). Low-abundance profiles were eliminated. In addition, if multiple ensemble IDs correspond to the same gene symbol, the one with the highest mean value was extracted as the expression level of parent gene.

Subsequently, corresponding clinicopathological features of BC patients in TCGA cohort, including the gender, age, histological type, history of neoadjuvant treatment, regional lymph nodes involvement (H&E stain), TNM stage, pathological stage, estrogen receptor (ER) status, progesterone receptor (PR) status, human epidermal growth factor receptor-2 (Her-2) status, and follow-up data, were also downloaded via “TCGAbiolinks” package and used for subsequent analysis. Therein, the Her-2 statuses of BC samples were firstly quantified by immunohistochemical (IHC) results, and we took an account of the results from fluorescence *in situ* hybridization (FISH) to determine the Her-2 status when the IHC diagnoses were missed or uncertain. The present study fully complies with the TCGA publication guidelines (Network, 2012).

The integrated methylation matrix files (GSE75067 and GSE72308) based on the platform GPL13534 were obtained from the Gene Expression Omnibus (GEO) database (https://www.ncbi.nlm.nih.gov/geo). Among these two datasets, the missing values in methylation profiles were also imputed via KNN algorithm. And the corresponding clinical data were obtained either by directly downloading the attached files from the item page in the GEO website (GSE75067) or retrieving and manually organizing if available (GSE72308).

### Study Population and Eligibility Criteria

The backbone of our study was comprised of three stages: discovery stage, training stage, and validation stage. In the discovery stage, we used paired samples (tumor/normal) from BC patients in the TCGA-BRCA project as discovery set (*n* = 91 pairs), which was used to identify the differential methylation CpG sites. In the training stage, a total of 654 BC patients from the entire TCGA cohort were enrolled as the training set with the following inclusion criteria: 1) female patients with a definitive diagnosis of BC, 2) without any neoadjuvant therapy, 3) with corresponding follow-up censoring data, 4) the follow-up time was no less than one month, and 5) with corresponding DNA methylation profiles. Furthermore, independent validations were conducted using two external GEO datasets in the validation stage. For validation set 1, the clinical and DNA methylation data were obtained from accession number GSE75067 (Holm et al., 2016). Only 180 BC patients were included in our analysis, after the removal of repetitions and samples with missing survival information. For validation set 2 (accession number: GSE72308), a subset of 237 BC patients (cohort 1 and cohort 2) treated with adjuvant therapies from 1995 to 2009 was included and the patients who had a history of neoadjuvant epirubicin monotherapy (TOP cohort) were excluded, as described by Jeschke et al. (2017).

### Identification of Differentially Methylated CpG Sites

On the basis of the discovery set of 91 BC patients who had both tumor and adjacent-normal tissues, genome-wide differential methylation CpG sites were identified using paired Student’s *t*-test. *p*-values were adjusted by Benjamini & Hochberg (BH) correction; the methylation differences were characterized by absolute differential methylation calculated as mean (βtumor) − mean (βnormal) and adjusted *p*-values. An absolute differential methylation of >0.4 combined with adjusted *p* < 0.05 was set as the significance threshold. Additionally, the diagnostic values of specific CpG sites were evaluated by receiver operator characteristic (ROC) curve.

### Construction and Validation of a CpG-Based Prognostic Model

In the training stage, the initial assessment of prognostic potential of each differential methylation CpG site was conducted using univariate Cox proportional hazard regression analysis, and those with *p*-values < 0.05 were selected for further analyses. Next, for solving the multicollinearity problem of highly correlated variables, we implemented the Cox regression model, with least absolute shrinkage and selection operator (LASSO) penalty, to reduce dimension (Friedman et al., 2010; Simon et al., 2011). Based on prognostic BC-specific CpG sites, which were significant in the univariate Cox regression analysis, a sub-selection of key biomarkers was determined by shrinkage of the regression coefficients via using a penalized weight to corresponding size. Finally, only the features with nonzero regression coefficient were extracted as the candidates for constructing prognostic signature. In our LASSO analysis, the “lambda.min” criteria and 10-fold cross validation were utilized to conduct penalty parameter tuning. Subsequently, we computed an epigenetic risk score for each BC patient using the following regression equation: $\text{risk score }=\;\sum_{j=1}^{n}Coef_{j}\beta_{j}$, with$Coef_{j}$indicating the coefficients derived from multivariate Cox regression model and$\beta_{j}$representing the methylation level (β value) of each CpG site. And the predictive performance of CpG-based risk score was assessed by time-dependent ROC curves with area under curve (AUC) values calculated (Blanche et al., 2013). Then we classified the BC patients in the training set into low- and high-risk subgroups based on the optimal cutoff value of risk scores computed using regression formula, which represented the point at which the Youden index (specificity + sensitivity - 1) reached a maximum value in 5-years ROC curve. Furthermore, the Kaplan-Meier survival analysis and log-rank test were applied to verify the classification performance of the model.

### Independence of CpG-Based Signature From Other Clinicopathological Parameters in TCGA

To further validate whether the prognostic value of established CpG-based signature is independent of traditional clinicopathological parameters (including age, histological subtype, regional lymph nodes metastasis, T stage, N stage, pathological stage, ER, PR, and Her-2 status) for BC patients, stratification Cox analyses were conducted in various stratified cohorts. Additionally, among 654 BC patients with survival information in the training set, 537 BC patients with relatively complete clinical information, including age, histological type, regional lymph nodes metastasis, T stage, N stage, pathological stage, ER, PR, and Her-2 status, were subjected to subsequent analyses. Univariate followed by multivariate Cox regression analyses were performed.

### Construction and Evaluation of a Predictive Nomogram

The independent clinicopathological covariates, identified by multivariate Cox regression analysis, were taken into consideration to assemble a nomogram, providing clinicians with a clinically relevant quantitative approach for predicting individualized survival probability in BC patients (Harrell et al., 1996; Zhang and Kattan, 2017). Additionally, the calibration plots were graphically depicted to evaluate the consistency between the predicted probability of derived nomogram and actual situation (Balachandran et al., 2015). And Harrell’s concordance index (c-index) was utilized to estimate the discrimination ability of the nomogram. Moreover, decision curve analysis (DCA) was performed to explore the clinical usefulness of the nomogram, further providing straightforward information concerning whether clinical decision-making based on a predictive model will do more good than harm, in contrast to abstract statistical concepts (Fitzgerald et al., 2015; Vickers and Elkin, 2016; Zhang et al., 2018d).

### Gene Set Enrichment Analysis of CpG-Based Signature

In order to identify crucial biological mechanisms related to the final CpG-based signature in BC carcinogenesis, we implemented GSEA analysis (version 4.0.2, http://software.broadinstitute.org/gsea/downloads.jsp) using the adjusted RNA-sequencing profiles (TPM normalization) for all transcripts (Subramanian et al., 2005; Reimand et al., 2019). Annotated gene set files were downloaded from Molecular Signatures Databases (MSigDB, http://software.broadinstitute.org/gsea/msigdb), and the “c2.cp.v7.0.symbols.gmt” were selected to perform the quantification of pathway activity (Liberzon et al., 2011; Liberzon et al., 2015). Enrichment *p*-values were evaluated based on 1,000 permutations and subsequently adjusted by BH correction for multiple testing to control FDR values. Therein, nominal *p*-value < 0.05 and FDR < 0.25 were set as significance threshold.

### Comprehensive Evaluation of TME in BC Patients

To decipher the role of established signature in modifying TME in BC patients, we comprehensively analyzed the association between the risk score and immunological characteristics within TME with respect to the below aspects. The immune score, stromal score, and tumor purity in BC samples were calculated by applying the Estimation of STromal and Immune cells in MAlignant Tumor tissues using Expression data (ESTIMATE) algorithm designed by Yoshihara et al. (2013). Thereafter, we applied the Microenvironment Cell Populations-counter (MCP-counter) algorithm, an estimator for robust quantification of absolute abundance of two stromal and eight immune cell populations by gene expression profiles, to perform the immune infiltration estimation in BC tissues (Becht et al., 2016). The infiltration levels of immune cells were derived using “MCPcounter” R package (version 1.1.0). To avoid the calculation error due to distinct inner algorithm, additional independent algorithms, including the Cibersort (with LM22 signature) and single sample Gene Set Enrichment Analysis (ssGSEA) methods, were utilized to quantify the infiltration levels of immune cells within TME (Chen et al., 2018; Zhang et al., 2018b). A list of 24 immune inhibitory checkpoint molecules, 122 immunomodulators and effector genes of particular tumor-infiltrating immune cells were collected from hand-curated screenings of literature (Auslander et al., 2018; Hu et al., 2021). Moreover, based on the ssGSEA algorithm, we profiled the enrichment scores of seven steps that composed the “anti-cancer immunity cycle,” including release of cancer cell antigens (Step 1), cancer antigen presentation (Step 2), priming and activation (Step 3), trafficking of immune cells to tumors (Step 4), infiltration of immune cells into tumors (Step 5), recognition of cancer cells by T cells (Step 6), and killing of cancer cells (Step 7) (Xu et al., 2018).

Furthermore, immune cytolytic score (CLS), defined as the log-average (geometric mean) of PRF1 and GZMA in TPM expression values, was also estimated to represent in silico measurement of immune infiltration, as provided by Rooney et al. (2015). Pan-cancer T cell inflamed score (TIS), as a weighted linear combination of scores from eighteen genes, which defines the pre-existing cancer immunogenicity and predicts response to anti-tumor immunotherapy, was also computed for each BC patient (Ayers et al., 2017). The stemness indices (mRNAsi) for BC patients were derived from the supplementary table by Tathiane et al., who measured the oncogenic dedifferentiation and were associated with Immune microenvironment contents and PD-L1 levels (Malta et al., 2018). Immunophenoscore (IPS), derived from a panel of immune-related genes belonging to four categories, namely effector genes, immunosuppressive genes, MHC-related molecules, and immunomodulators, represents the cancer antigenomes and immunogenicity (Charoentong et al., 2017). The IPSs of BC patients were obtained from The Cancer Immunome Atlas (TCIA) (https://tcia.at/home). Additionally, Vésteinn Thorsson and his colleagues identified and characterized six immune subtypes that encompass multiple tumor types, with distinct intratumoral immune states and immune response patterns impacting prognosis (Thorsson et al., 2018). In our present work, we also classified the BC samples into different immune subtypes using the ImmuneSubtypeClassifier R package (https://github.com/Gibbsdavidl/ImmuneSubtypeClassifier).

### External Validation of CpG-Based Signature

To evaluate the robustness and practical application of the CpG-based signature in predicting the OS probability for BC patients, the performance of this prognostic signature was further validated based on two independent cohorts. With the consistent regression formula and cutoff value of CpG-based signature derived from the training set, we also stratified the BC patients in validation set 1 (*n* = 180) and set 2 (*n* = 237) into low-risk and high-risk subgroups, respectively. And we applied Kaplan-Meier survival analysis to compare the OS rates of these subgroups. Additionally, time-dependent ROC curves were conducted to investigate the prognostic efficacy of the model in these external cohorts.

### Correlation of Epigenetic Risk Score With Mutations and Copy Number Aberrations

Somatic mutation profiles and copy number variation data of BC patients were downloaded from TCGA repositories and classified into two distinct groups according to the established risk score. Therein, the driver mutation genes were analyzed and visualized using “maftools” R package (Mayakonda et al., 2018), and significant amplifications or deletions among the whole genomic region were identified via GISTIC 2.0 software (Mermel et al., 2011), respectively. Additionally, tumor mutation burden (TMB) of each patient was calculated via counting the total number of non-synonymous mutation events per megabase within whole genome (38 Mb was utilized as the estimate of the exome size). The burden of copy number losses or gains was defined as the total number of genes with copy number aberrations at the arm and focal levels (Sia et al., 2017).

### Therapeutic Response Prediction in BC Patients

To further investigate the potential therapeutic properties for BC patients with distinct prognoses, we collected a series of therapeutic signatures, including the oncogenic pathways shaping non-inflamed TME and gene signatures for targeted therapies and radiotherapy. Therein, GSVA algorithm was implemented to calculate the corresponding enrichment scores, quantifying the possibility for specific therapeutic response for each patient (Hänzelmann et al., 2013).

The programmed cell death 1 (PD-1) and cytotoxic T-lymphocyte associated protein 4 (CTLA-4) are involved in tumor immune evasion. We combined the unsupervised subclass mapping method (SubMap, https://cloud.genepattern.org/gp/) and pretreatment genomics to predict the clinical responses to immune checkpoint blockade for distinct risk BC patients (Hoshida et al., 2007; Lu et al., 2019).

Based on the Genomics of Drug Sensitivity in Cancer (GDSC) database, a large public pharmacogenomics repository, we predicted the potential drug resistance to six commonly used chemotherapeutic agents, namely, cisplatin, gemcitabine, docetaxel, paclitaxel, etoposide and vinorelbine. R package “pRRophetic,” implements ridge regression and 10-fold cross validation to help in estimating the half-maximum inhibitory concentration (IC50) of each sample, according to GDSC training cell lines (Geeleher et al., 2014).

The Broad Institute’s connectivity Map build 02 database (CMap, https://portals.broadinstitute.org/cmap/) was a comprehensive resource for investigating relationships among biomarkers, diseases, and therapeutics (Qu and Rajpal, 2012). To screen potential candidate compounds targeting crosstalk against CpG sites derived from established signature, we calculated the differential expression gene (DEG) list between low- and high-risk patients and selected the top 300 to query the CMap database. Furthermore, the shared mechanisms of actions (MoA) among perturbagens were revealed via specific analysis within CMap tools (https://clue.io/) (Subramanian et al., 2017).

### Statistical Analysis

Shapiro-Wilk normality test was utilized to test the normality of variables. For pairwise comparisons, statistical significance for non-normally distributed variables was estimated by wilcoxon test (Mann-Whitney U test), whereas normally distributed variables were analyzed by Student’s *t*-test. The significance of correlations between variables was computed by Spearman or Pearson correlation analysis. Contingency tables were analyzed by two-sided Fisher’s exact tests. All the statistical analyses were conducted using R (version 3.5.3, https:// www.r-project.org/) software and a two-tailed *p*-value < 0.05 was considered statistically significant. Hierarchical cluster analysis using the Euclidean distance method to calculate dissimilarity structure was graphically explored with the “pheatmap” package. The LASSO-penalized Cox regression model was performed with “glmnet” package. ROC curves were derived using “pROC” and “PRROC” package, whereas the estimation of time-dependent ROC curves and AUC values of censored survival data were computed using “timeROC” package. The nomogram and calibration plots were generated via “rms” package, and the c-index was calculated by “Hmisc” package. The DCA analyses were performed with the “dca.R” source code.

## Results

### Selection of Cancer-Specific CpG Sites in BC Samples

**FIGURE 1:**
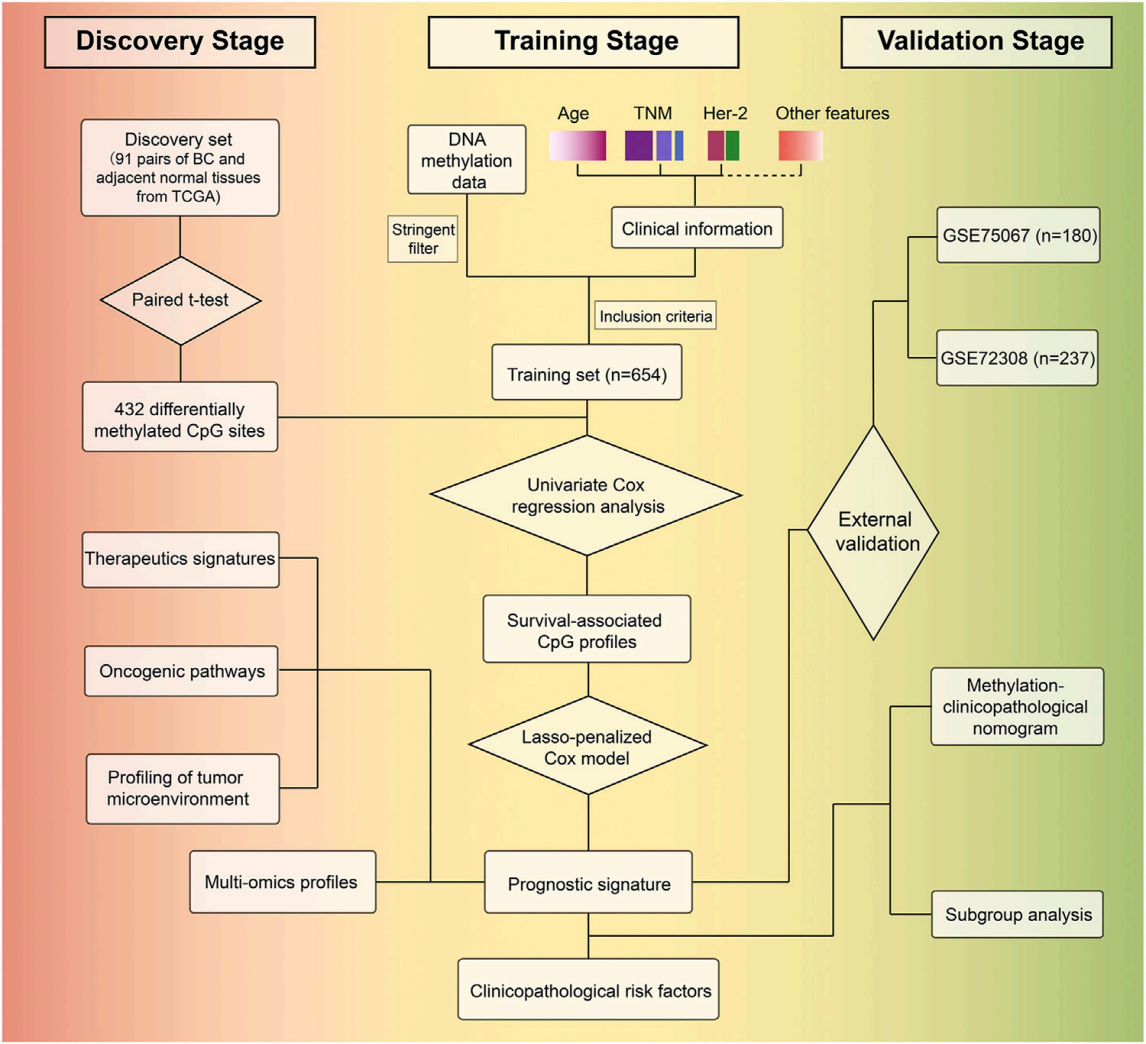
Study overview for profiling the prognostic signature based on large-scale DNA methylation data.

**FIGURE 2:**
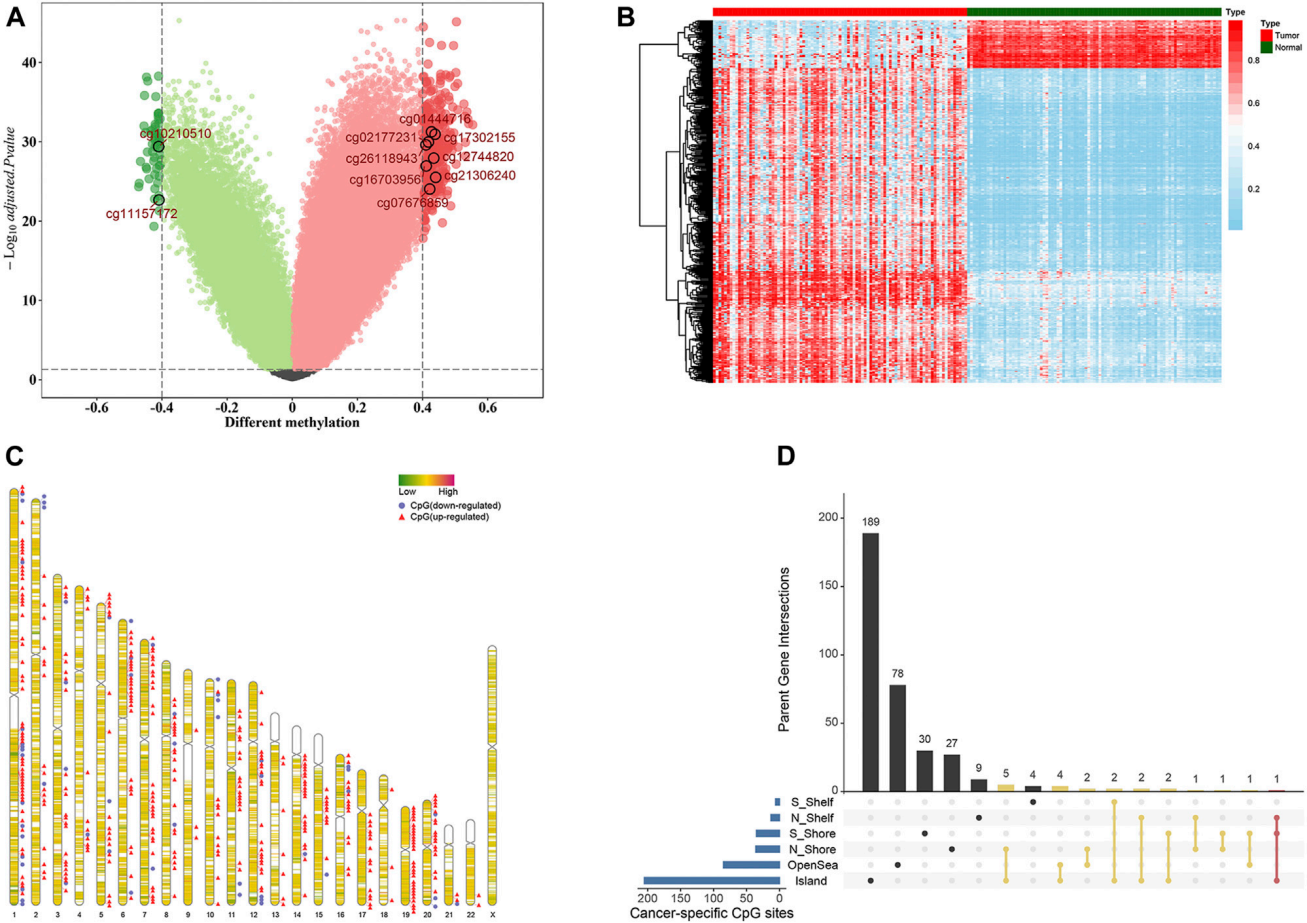
Selection of differential methylation CpG sites in the discovery stage (*n* = 91 pairs). **(A)** Volcano plot was generated based on the differential methylation in combination with adjusted *t*-test *p*-values. The *x*-axis represents the differential methylation that the average methylation level in tumor tissues versus average value in adjacent normal tissues for each CpG site, while the *y*-axis represents the negative log10 transformation of adjusted *p*-values for each comparison. The vertical lines represent an absolute value of differential methylation of 0.4, and the horizontal lines indicate adjusted *p*-value = 0.05. A total of 432 differentially methylated CpG sites with statistical significance were identified. Among them, 375 were up-regulated (red dots) and 57 were down-regulated (green dots). **(B)** Heatmap showing methylation of 432 CpG sites in paired tumor samples and adjacent normal tissues. **(C)** Idiogram visualizing the genome-wide information of differential methylation CpG sites. **(D)** Upset plot depicting the region-level island-based distribution within parent genes of these 432 CpG sites.

We developed the model for the prognosis prediction of BC patients in three stages: discovery, training, and validation stage. The study flowchart is depicted in Figure 1. After a series of stringent filtering, a total of 308786 CpG sites were chosen as the background for the selection of candidate CpG sites. Firstly, in the discovery stage, volcano plot analysis (absolute differential methylation >0.4 and adjusted *p* < 0.05) identified 432 differentially methylated CpG sites (Figure 2A). Therein, 375 CpG sites were found to be hypermethylated and 57 CpG sites were hypomethylated, which corresponded to 308 and 54 genes, respectively. With regard to these cancer-specific CpG sites, hierarchical cluster analysis successfully segregated the 91 pairs of tumor/normal samples into two distinct clusters (Figure 2B). Then idiogram was used to map and visualize the genome-wide information of these particular biomarkers into chromosomes (Figure 2C). Additionally, Upset plot was depicted to investigate the region-level island based distribution of these CpG sites across different genomic regions (Figure 2D). And it was found that the majority of these CpG sites were significantly enriched within the island and opensea, whereas only a few of them were located in the N shelf and S shelf.

### Construction of a CpG-Based Signature and Evaluation of Its Predictive Ability in the TCGA BC Cohort

**FIGURE 3:**
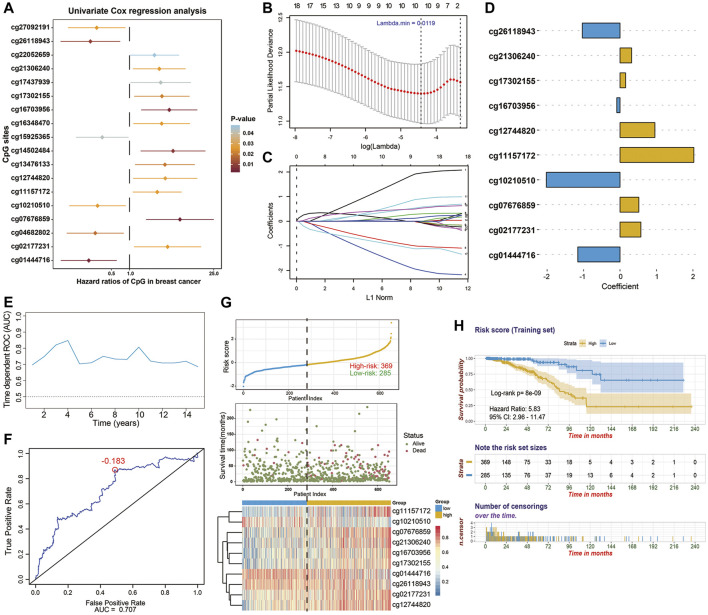
Identification of 10-CpG-based prognostic signature in the training stage (*n* = 654). **(A)** Forest plot which depicts the 18 significantly survival-associated CpG sites (*p*-value < 0.05) among the cancer-specific methylation locus in the training set. Unadjusted hazard ratios (boxes) and 95% confidence intervals (horizontal lines) of univariate Cox regression analyses are presented in the plot. *p*-values are indicated by the color scale by the right side. **(B)** Selection of tuning parameter (lambda) in the LASSO-penalized Cox model via 10-fold cross validation in the training set. The partial likelihood deviance (*y*-axis) from the cross-validation procedure of LASSO regression is plotted as a function of log(lambda) (lower *x*-axis). Dynamically changing number along the upper *x*-axis indicates the average number of predictors. Dashed vertical lines from right to left define the logarithm of the optimal value of lambda via “1-fold standard error” and “minimum” criteria, respectively. **(C)** The shrinkage procedure of regression coefficients of predictors in **(B)**. The optimal lambda value of 0.0119 was chosen based on the “minimum” criteria and 10-fold cross validation. The 10 resulting predictors with nonzero coefficients were subselected as candidates for model construction. And the numbered lines represented the corresponding CpG sites in *y*-axis of the **(A)** from top to bottom, respectively. **(D)** Coefficients of CpG sites derived from the multivariate Cox regression analysis, which were used for calculating the risk score. **(E)** The performance of the model in training set. Dynamically time-dependent ROC curves with AUC values estimated from 1 year to 15 years. **(F)** The 5-years ROC curve analyses for the risk score derived from the 10-CpG-based signature. The red dot represents the corresponding optimal cutoff value (−0.183), which were calculated based on the Youden index. **(G)** The distribution of patients’ survival status ranked by corresponding risk score, the methylation pattern of corresponding CpG sites included in final signature, **(H)** Kaplan-Meier curves of overall survival between low- and high-risk patients stratified by 10-CpG-based signature in the training cohort. Log-rank test, *p*-value < 0.0001.

In the training stage, 654 BC patients with integrated methylation profiles and clinical information were retrospectively analyzed in depth, among which 66 (10.1%) died and the median survival time was 115.7 months. The median follow-up time of these BC patients was 17.3 months (range, 1.0–235.6 months). For the entire cohort, the 1-, 3-, 5-, and 8-years OS rates were 98.3, 91.5, 80.4, and 61.5%, respectively. To investigate the prognostic values of these cancer-specific biomarkers in BC patients, univariate Cox regression analyses were performed. As results, 18 CpG sites, the methylation levels of which were significantly correlated with OS (*p* < 0.05), were predominantly identified as the candidates (Figure 3A). To screen out the CpG sites with the greatest potential prognostic values, we then applied a LASSO-penalized Cox regression model and narrowed down a methylation-based signature for BC patients in our training cohort (Figures 3B,C). Of these 18 candidate CpGs, 10 were finally identified, namely cg11157172, cg21306240, cg10210510, cg02177231, cg16703956, cg26118943, cg17302155, cg12744820, cg01444716 and cg07676859, mapped to PRDM16, EPHA10, COL9A2, TBX15, SLC6A3, SNX18, PRDM13, OLIG3, BTBD3, and SSTR4, respectively. Using coefficients weighted by multivariate Cox regression model (Figure 3D), an epigenetic score was calculated for each BC patient based on the individualized methylation levels of corresponding CpG sites as follows:

[2.0266 × Methylation level of cg11157172] + [0.3172 × Methylation level of cg21306240] + [(−2.0224) × Methylation level of cg10210510] + [0.5711 × Methylation level of cg02177231] + [(−0.0887) × Methylation level of cg16703956] + [(−1.0346) × Methylation level of cg26118943] + [0.1536 × Methylation level of cg17302155] + [0.9559 × Methylation level of cg12744820] + [(−1.1586) × Methylation level of cg01444716] + [0.5151 × Methylation level of cg07676859]

Using the Youden index to generate the optimal cutoff value of the risk score, we assigned 369 BC patients (56.4%) with a derived risk score > −0.183 to a high-risk subgroup and others (43.6%) to a low-risk subgroup (Figure 3G). Kaplan-Meier survival analysis (Figure 3H) also revealed the risk of high-risk patients was 5.83-fold higher than that in the low-risk group (95%CI, 2.96–11.47; *p* < 0.0001). Next, time dependent ROC curves and C-index were performed to investigate the predictive efficiency of this established model (Figures 3E,F). The resulting AUC values of ROC curve remained above 0.7 even up to 15 years and C-index was 0.743 (95%CI, 0.675–0.811), indicating that our 10*-*CpG-based signature had superior predictive efficiency. The distribution of risk score, patients’ survival status, and methylation patterns of corresponding CpG sites are shown in Figure 3G.

### The CpG-Based Signature Is Independent of Conventional Clinicopathological Features

In order to investigate the prognostic value of 10*-*CpG-based signature in stratified cohorts, we classified BC patients into various subgroups and next performed stratification Cox analyses for the entire cohort according to available clinicopathologic characteristics. As expected, our signature was able to classify patients with distinct prognoses, thus confirming its robustness for independently predicting BC prognosis (Supplementary Figure S1). Furthermore, 537 samples with relatively complete clinical information within TCGA-BC cohort were subjected to verify whether our signature was an independent predictive factor for the prognosis of BC patients. The results of univariate analyses suggested that age, ER status, PR status, N stage, positive lymph nodes status, pathological stage, and risk score derived from 10*-*CpG-based signature were all remarkably correlated with OS of BC patients (Supplementary Figure S2A). Hence, these risk factors were included to perform a multivariate Cox analysis and our results indicated the CpG-based signature was an independent prognostic factor when adjusted by those factors (Supplementary Figure S2B). Based on the PAM50 subtype classification of BC, the signature also shows its robust prognostic ability in basal-like and luminal subtype (Supplementary Figures S2C,D).

### Construction and Assessment of a Novel Methylation-Clinicopathological Nomogram

**FIGURE 4:**
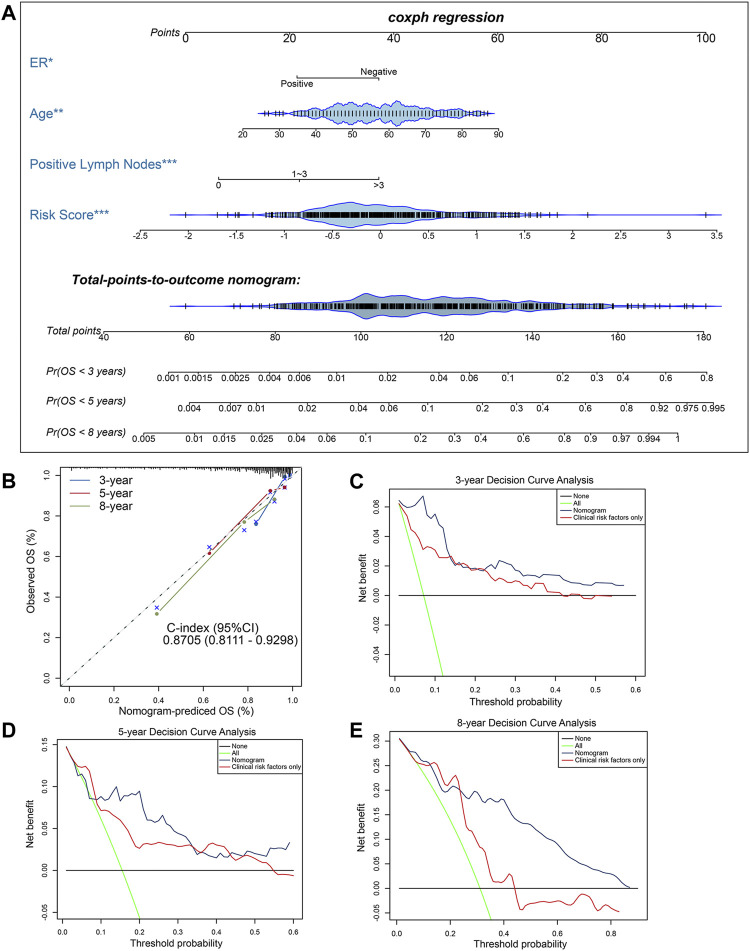
Construction and evaluation of a clinical predictive model. **(A)** The methylation-clinicopathologic nomogram for predicting the 3-, 5- and 8-years overall survival probability for BC patients, which was developed in the TCGA cohort, with ER status, age, positive lymph nodes status and 10*-*CpG-based signature incorporated. **(B)** Calibration curves and C-index (95% CI) of the nomogram in TCGA cohort. The calibration plot depicts the calibration of the nomogram in term of agreement between observed and predicted 3-, 5- and 8-years clinical outcomes. The dashed 45-degree line indicates the ideal situation, and the dotted lines indicate the predictive performance of the model. **(C**–**E)** Decision curve analysis of the nomogram in TCGA cohort for evaluating the clinical usefulness in **(C)** 3 years, **(D)** 5 years and **(E)** 8 years. The blue line represents the CpG-clinicopathologic nomogram. The red line represents the model which integrated clinical risk factors only. The black line represents the treat-none scheme. And the green line represents the treat-all-patients scheme.

To further develop a clinically applicable approach for predicting OS probability in BC patients, a secondary multivariate Cox regression analysis that integrated the identified independent risk factors, consisting of age, ER status, positive lymph nodes status and risk score, was performed and depicted as an inclusive nomogram (Figure 4A). It substantiated that the risk score contributes the greatest weight to the total points, whereas other clinicopathological features contribute much less, which were consistent with results from previous multivariate regression analyses. The C-index of our nomogram for OS prediction reached 0.8705 with 1,000 bootstrap replicates (95% CI: 0.8111–0.9298), indicating a favorable discriminatory performance of derived nomogram. And the results of calibration curves showed that the bias-corrected lines were close to the ideal line (45-degree line), indicating good agreement between the predicted and actually observed outcomes (Figure 4B). Moreover, we performed DCA analysis to evaluate the real-world clinical usefulness of the inclusive nomogram by quantifying the net benefits against a range of threshold probabilities. The DCA results of the nomogram are presented (Figures 4C–E), revealing that the prognosis-related treatment decision-making based on our methylation-clinicopathological nomogram could add more net benefit than treat either none or all patients if the probability threshold for doctors or patients does not exceed 60%. Besides, the clinical usefulness of our nomogram significantly overwhelmed the conventional clinicopathological factors. Consequently, these findings suggested that our nomogram was an optimal prognostic model for predicting both short-term and long-term survival probability in BC patients.

### External Validation and Evaluation of 10-CpG-Based Prognostic Signature in the GEO BC Cohorts

**FIGURE 5:**
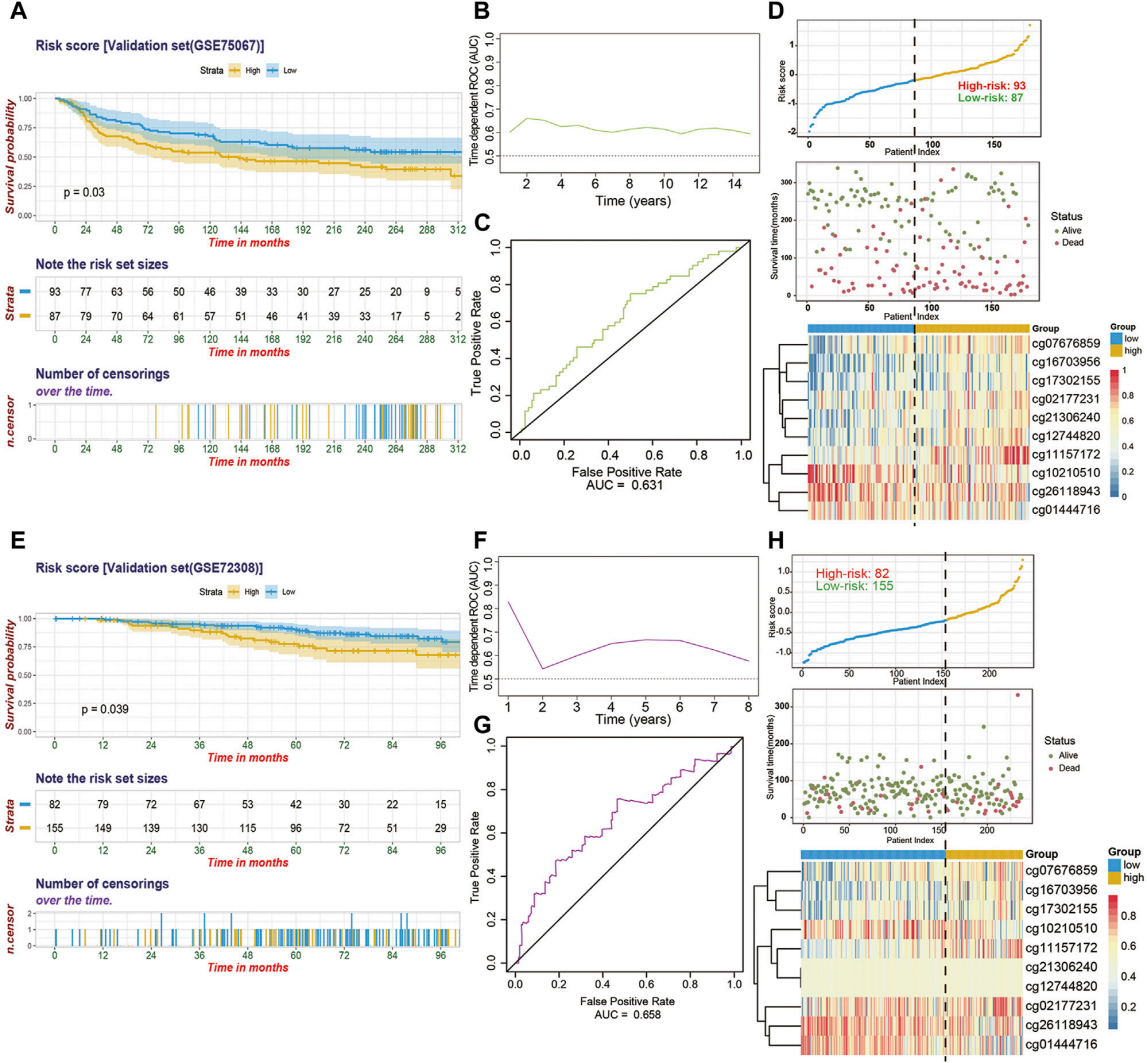
The performance of the 10*-*CpG-based signature in validation set 1 (GSE75067) and validation set 2 (GSE72308). **(A)** Kaplan-Meier curves depicting the significant difference of overall survival between low- and high-risk patients stratified by 10*-*CpG-based signature in GSE75067 cohort. Log-rank test, *p*-value = 0.03. **(B)** Time-dependent ROC curves with AUC values estimated of the prognostic signature in GSE75067 cohort from 1 year to 15 years. **(C)** The 5-years ROC analysis of the prognostic signature in GSE75067 cohort. **(D)** The relationship among the distribution of patients’ risk score (upper), survival status (middle), and the methylation pattern of the 10 particular CpG sites (bottom) in GSE75067 cohort is shown. The Kaplan-Meier survival analysis **(E)**, time-dependent ROC curves with AUC values calculated **(F**–**G)** and risk score analysis **(H)** for the 10*-*CpG-based signature in GSE72308 cohort. Log-rank test, *p*-value = 0.039.

To substantiate the stability of final 10*-*CpG-based signature, external analyses were performed in two external validation cohorts: GSE75067 and a subset of GSE72308. Utilizing the same risk score formula and cutoff value obtained from the training cohort, the BC patients in the GSE75067 dataset were categorized into a high-risk (51.7%) subgroup and a low-risk (48.3%) subgroup. Consistent with the outcomes of TCGA-BC cohort, the patients who were assigned to the high-risk subgroup had significantly worse OS than low-risk BC patients (Figure 5A; HR, 1.576; 95%CI, 1.041–2.385; *p* = 0.03), demonstrating the robustness and clinical applicability of the established signature across different cohorts. And time-dependent ROC analysis revealed the CpG signature had favorable efficacy for predicting both short-term and long-term OS, with AUC value maintaining above 0.6 from 1 year to 15 years (Figures 5B,C). The distribution of risk score and methylation patterns of 10 particular CpG sites are shown (Figure 5D). Furthermore, similar analyses showed that the CpG signature could successfully divide 155 BC patients (65.4%) into low-risk subgroup and 82 patients (34.6%) into high-risk subgroup in term of OS (HR, 1.876; 95%CI, 1.023–3.44; *p* = 0.039) in the GSE72308 cohort (Figure 5E). Similarly, the CpG signature maintained its discriminative power for prognosis prediction spanning from 1 year to 8 years (Figures 5F,G). Additionally, the risk score distribution, survival status, and methylation patterns of 10 CpG biomarkers are displayed (Figure 5H). Taken together, these results suggested that our 10*-*CpG-based signature is robust for differentiating BC patients with a favorable or poor prognosis, which may possess the pivotal mechanism underlying the BC carcinogenesis, progression, and metastasis.

### Decipher the Diagnostic Values of These CpG Sites in BC

These identified ten CpG sites were significantly differentially methylated in paired tumor/adjacent-normal tissues (Supplementary Figure S3). ROC curves revealed that these CpG could individually or jointly show diagnostic value for BC in high efficacy in discovery set (Supplementary Figure S4) and entire TCGA-BC dataset (Supplementary Figure S5). Additionally, Spearman correlation analyses were performed to estimate the prospective methylation and expression quantitative trait loci (meQTL) relationships (Supplementary Figure S6). As results, hypermethylation of CpG sites (cg01444716, cg02177231, cg26118943, and cg07676859) inhibited expression of corresponding gene (BTBD3, TBX15, SNX18, and SSTR4), while hypermethylation (cg11157172, cg21306240, and cg10210510) was positively correlated with parent gene (PRDM16, EPHA10, and COL9A2) expression. However, methylation levels of three other CpG sites (cg12744820, cg16703956, and cg17302155), which were located in 1st exon of OLIG3, TSS1500 of SLC6A3, and gene body of PRDM13, respectively, did not display any correlation with the gene expression.

### Altered Biological Processes and Pathways in High- and Low-Risk BC Patients

**FIGURE 6:**
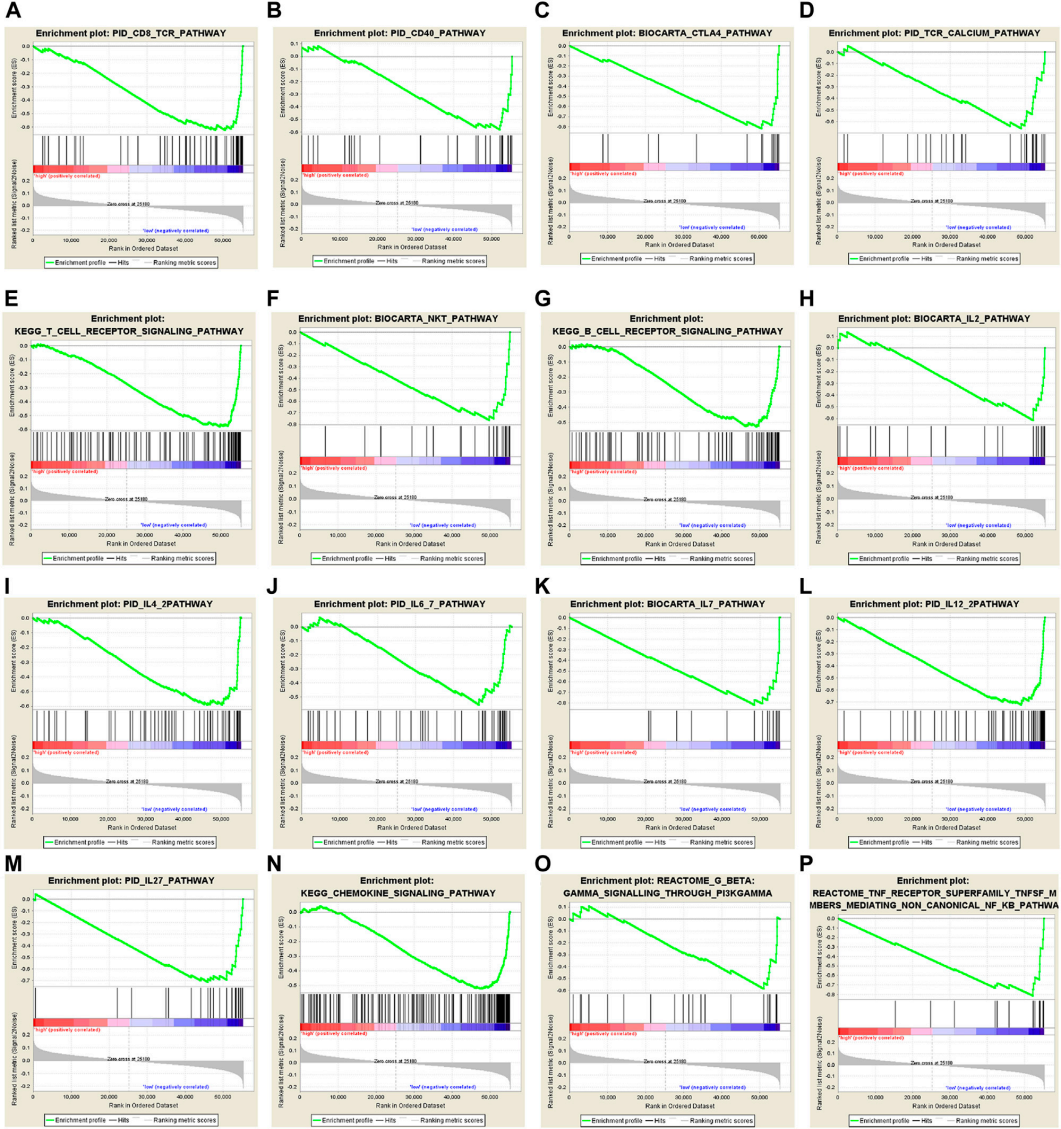
Gene set enrichment analysis (GSEA) delineates the significantly enriched pathways which are negatively correlated with 10*-*CpG-based signature using the “c2.cp.v7.0. symbols” gene set, downloaded from the MSigDB database, as the reference. And the significance threshold was set at nominal *p*-value < 0.05 and FDR < 0.25.

The strong risk stratification ability of our 10*-*CpG-based signature for BC patients could be attributed to their potential regulation in tumor development or metastasis. Therefore, GSEA analysis was performed to elucidate the association between potential biological phenotypes and our prognostic signature. In the GSEA enrichment results, we noticed that genes highly expressed in the high-risk patients were significantly enriched in multiple cancer-related pathways, such as Notch4 signaling pathway (normalized enrichment score, NES = 1.937, size = 53), cell cycle regulation (NES = 1.866, size = 85), stabilization of P53 (NES = 1.806, size = 55), regulation of DNA damage checkpoints (NES = 1.917, size = 66), and apoptosis (NES = 1.951, size = 52) (*p* < 0.05 and FDR < 0.25) (Supplementary Figure S7). In contrast, the CpG-based model was negatively correlated with immune relevant behaviors, including alteration of immune checkpoint molecules, activation of immune cell signaling pathways, and Interleukin-related signatures (Figure 6), further demonstrating low-risk BCs were characterized as an enhanced immune phenotype. Therefore, it could be speculated that our CpG-based signature might serve as an interface between epigenetic modification and immune activities. In summary, our GSEA analysis implied that these CpG sites play crucial roles in mammary carcinogenesis via the above-referenced biological processes and their functional dysregulation subtly affected the OS of BC patients.

### The Risk Score Shapes a Non-inflamed TME in BC

**FIGURE 7:**
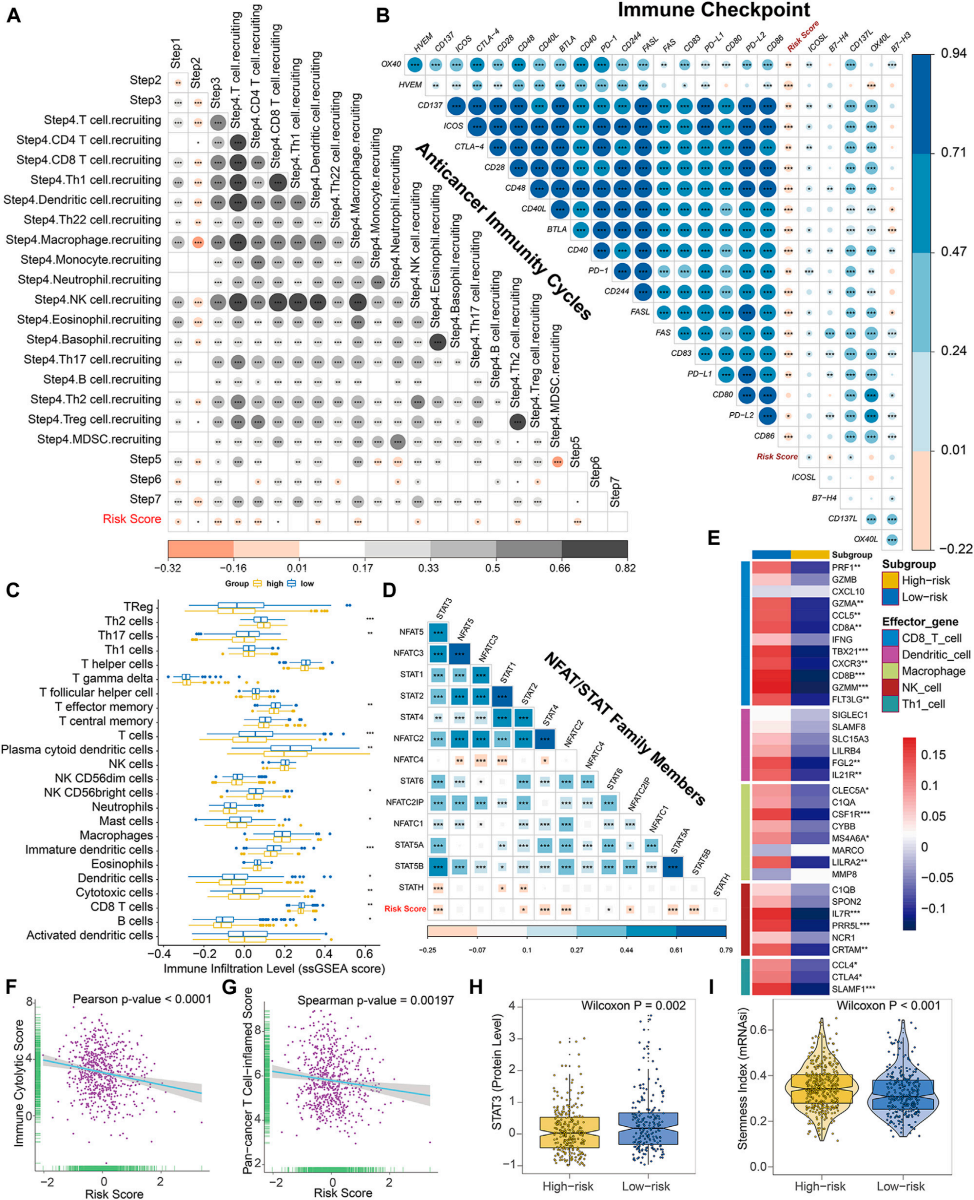
The epigenetic risk score shapes a non-inflamed TME in BC. **(A)** Correlation between risk score and enrichment scores of various steps of the anticancer immunity cycle. **(B)** Correlation between risk score and expression levels of 24 curated inhibitory immune checkpoints. **(C)** Differences in the infiltration levels of 24 immune cells (quantified by ssGSEA algorithm) between low- and high-risk subgroups. **(D)** Correlation between risk score and mRNA levels of NFAT and STAT family members. **(E)** Differences in expression levels (average z-scores) of the effector genes of tumor-associated immune cells between low- and high-risk patients. **(F)** Pearson correlation between immune cytolytic activities and risk score. **(G)** Spearman correlation between pan-cancer T cell inflamed signature and risk score. Both T cell inflamed score and immune cytolytic activities are positively correlated with clinical response to anti-cancer immunotherapy. **(H)** Differences in protein expression of STAT3 between low- and high-risk patients based on the reverse-phase protein array analysis. **(I)** Differences in stemness index (mRNAsi) between low- and high-risk patients. The statistical significance of pairwise correlations or comparisons is annotated with asterisks (*p < 0.05; **p < 0.01; ***p < 0.001).

Considering that a vast number of immunological pathways were downregulated in high-risk BC patients, we sought to explore the intrinsic correlation between the TME and our established risk scores to elucidate the immunogenicity of each risk subgroup. To execute this, we first evaluated the correlation between the risk score and seven steps of cancer-immunity cycle, which conceptualized the anti-cancer immune response. Overall, activities of the majority of the steps in the cycle were negatively correlated with our prognostic signature (Figure 7A), including the release of cancer cell antigens (Step 1), cancer antigen presentation (Step 2), priming and activation (Step 3), trafficking of immune cells to tumors (The recruiting of Th1 cell, dendritic cell, macrophage, NK cell, Th17 and Th2 cell, Step 4), and infiltration of immune cells into tumors (Step 5). Subsequently, for high-risk patients, the reduced activities of these steps may also impede the infiltration of tumor-associated immune cells and promote the formation of non-inflamed TME.

Thereafter, using the ESTIMATE algorithm in combination with the corresponding transcriptome profiles, we evaluated the differences in the TME between high- and low-risk BC patients, comprised of immune cell infiltration level (immune score), stromal cell content (stromal score), and tumor purity. Based on this algorithm, immune scores ranged from −1337.749 to 3747.411, and stromal scores were distributed between −2142.388 and 2125.969, respectively. And we found that both the immune and stromal scores were significantly elevated in low-risk subtype while an opposite trend was revealed when comparing the tumor purify of different risky subgroups (Supplementary Figures S8A–C), suggesting the risk score may reflect cancer immunogenicity in BC.

Consistently, the risk score was found to be mutually exclusive with a majority of immune checkpoint inhibitors, such as PD-1, PD-L1, CTLA-4, and so on (Figure 7B). Theoretically, the low abundance of checkpoint molecules was always indicative of resistance to immune checkpoint blockade. Moreover, the risk score was negatively correlated with a vast majority of immunomodulators (Supplementary Figure S9). Notably, a majority of MHC molecules were significantly downregulated in high-risk BC subgroup, suggesting potential regression on the capacity of antigen presentation and processing within these patients. Several critical chemokines, such as CCL19, CCL21, CCL22, XCR1, and paired receptors CCR7, CCR4, XCL1, and XCL2, were also negatively correlated with risk score. These chemokines and receptors could induce the recruitment of tumor-infiltrating immune cells such as CD8^+^ T cells and regulatory T cells (Treg). These outcomes indicated our risk score may exert a wide range of effects upon anticancer immune response.

Furthermore, for reducing calculation errors caused by different inner algorithms and marker gene sets, several independent methods, including ssGSEA, MCP-counter, and Cibersort, were applied to infer the infiltration levels of immune cells among BC patients using bulk RNA-seq data. Consistently, the majority of immune subpopulations within TME exhibited reduced infiltration in high-risk BC patients, indicating an immune-deserted TME in high-risk subgroup (Figure 7C, Supplementary Figures S8D–K, S10B). Of note, the infiltration levels of dendritic cells and CD8^+^ T cells, which induce antigen presentation and immune activation in tumors, were significantly lower in the high-risk subgroup based on these three methods. Both MCP-counter and Cibersort revealed that the infiltration level of macrophage was significantly reduced in high-risk patients. In addition, the high-risk subgroup exhibited downregulation of the effector genes of these tumor-infiltrating immune cells (Figure 7E). Additionally, the ssGSEA algorithm also detected that a wide range of immune cells was significantly decreased in high-risk BC subgroup, including T cells, Th17 cells, T effector memory cells, NK cells, mast cells, and B cells. We also found that cancer-associated fibroblasts were significantly downregulated in low-risk subtype. In addition, the risk score was negatively correlated with the gene signature of Th1 cells (Figure 7E). Collectively, for high-risk BC patients, the coordination role of these methylation biomarkers may restrain recruitment of tumor-associated infiltrating immune cells, transforming the inflamed surroundings into a non-inflamed TME.

Additionally, we observed that mRNA levels of STAT and NFAT family members, such as STAT3, STAT4, STAT5, and NFATC2, were also negatively correlated with our risk score (Figure 7D). The reverse-phase protein array data downloaded from cbioportal (http://www.cbioportal.org/) also validated that the protein level of STAT3 was significantly lower in high-risk patients compared with low-risk ones (Figure 7H). Given the evidence that NFAT and STAT family are well-known transcriptional regulators of immune checkpoints in T cells, these data indicated that DNA methylation biomarkers promote the downregulation of immune checkpoints synergistically in BC, potentially through the inhibition of NFAT/STAT signaling pathways.

To further authenticate our conjecture, we used CLS computed by Rooney et al., revealing the high-risk subgroup tends to exhibit downregulation of immune cytolytic activity (Figure 7F, Supplementary Figure S8L). The pan-cancer TIS is positively correlated with clinical response to anticancer immunotherapy and defines the pre-existing inflammatory status in TME. As expected, the risk score is negatively correlated with the T cell inflamed signature and the majority of genes within this signature (Figure 7G, Supplementary Figure S10A), further confirming its roles in shaping non-inflamed TME. And high-risk BC patients showed higher cancer stemlike indices (Figure 7I), indicating a potential negative regulation between stemness and anti-tumor immunity. Moreover, IPS was known as a superior immune response molecular marker to quantify the intratumoral immune landscape and predict response to immune checkpoint blockade therapy spanning multi-types of tumors. Of note, the epigenetic risk score was significantly negatively correlated with IPS (Supplementary Figure S11A) and the IPS, IPS-PD1-PD-L1-PD-L2, IPS-CTLA4, and IPS-PD1-PD-L1-PD-L2-CTLA4 scores in the high-risk subgroup were significantly lower compared with low-risk subgroup (Supplementary Figure S11B), indicating a less immunogenic phenotype and less sensitivity to immune checkpoint blockade treatment in high-risk patients. In addition, we found that in both the pre-defined low- and high-risk BC patients, C1 (Wound Healing) and C2 (IFN-γ Dominant) immune subtypes were mostly enriched with absence of C5 (Immunologically Quiet), which were in accordance with results from Thorsson et al. It was noteworthy that the high-risk subgroup had considerably more C1 and C2 subtypes characterized by high proliferation rate and poor prognosis, whereas low-risk ones occupied a higher percentage of C3 (Inflammatory) and C6 (TGF-β Dominant) subtypes with a high inflammatory signature and lymphocytic infiltrate (Supplementary Figure S11C). Thus, the distribution of immune subtypes between low- and high-risk patients was consistent with the prognosis and immunogenicity of each subgroup.

To further explore the contribution of each CpG site in shaping non-inflamed TME, we performed the correlation analyses between methylation levels of biomarkers and adverse indicators of modifying non-inflamed TME, including TIS, CLS, and IPS. As shown in Supplementary Figure S11D, the methylation level of cg102110510 was significantly positively correlated with TIS, CLS, and IPS, indicating its negative role in modifying a non-inflamed TME, whereas the epigenetic modification of cg21306240, cg17302155, cg16703956, cg12744820, and cg022177231 was significantly positive correlated with shaping non-inflamed TME in BC.

In summary, these results indicated that compared with its counterparts, BC patients who were divided into high-risk subtype via 10*-*CpG-based signature have a distinct immune phenotype, characterized by lower immune activation and being less efficacious to immunotherapies. Hence, a prior treatment option for BC with higher risk score was to transform a non-inflamed TME into an inflamed microenvironment, consequently triggering anti-cancer immune response.

### The Epigenetic Risk Score Is Associated With Distinct Genomic Imprints

**FIGURE 8:**
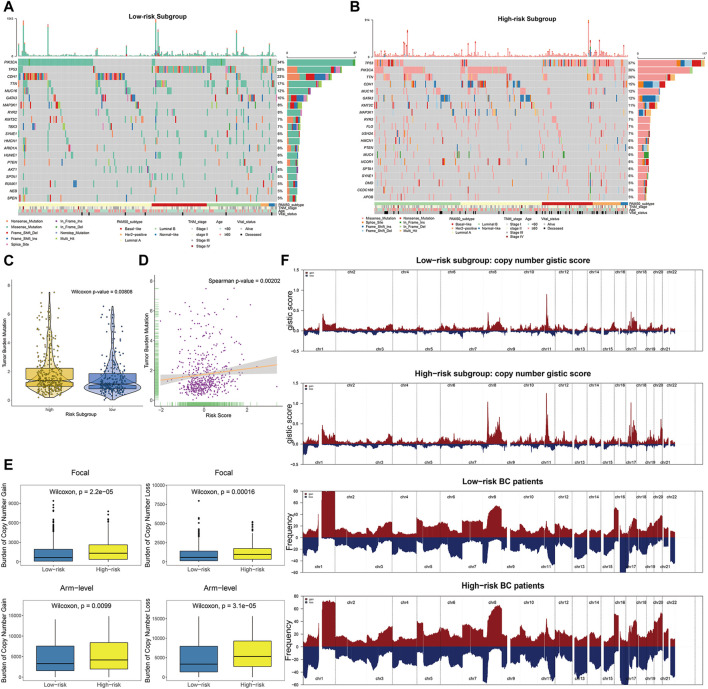
The landscape of mutational signatures and copy number alterations across distinct prognostic subgroups. **(A**–**B)** The oncoPrint depicting top 20 significantly mutated genes in the BC subsets stratified by risk scores, with corresponding clinicopathological characteristics of each subgroup annotated as below. **(C)** TMB difference in the high-risk and low-risk subgroups. Wilcoxon test, *p* = 0.00808. **(D)** Scatter plot depicting the positive correlation between TMB and risk scores in TCGA BC cohort. Spearman correlation between risk scores and mutation load is shown (*p* = 0.00202). **(E)** High-risk BC patients divided by risk scores showed significantly higher burden of gains and losses, both focal (upper panels) and arm levels (lower panels). **(F)** Composite copy number profiles (gistic scores or altered frequency) for low-risk and high-risk patients, with gains in dark red and losses shown in midnight blue. The copy number segments are placed according to corresponding chromosomal locations.

To explore the intrinsic correlation between established risk score and specific genomic alterations in BC, we performed multi-omics data analyses, including somatic mutation and copy number variation analysis using the corresponding TCGA profiles. In terms of somatic mutation analysis, patients in the high-risk subgroup had significantly higher somatic mutation burdens in TP53, KMT2C, and GATA3 (Figures 8A,B), which have been shown to play crucial roles in the carcinogenesis of multiple tumors. Additionally, patients in the high-risk subgroup showed a significantly higher overall TMB than low-risk ones (Wilcoxon-test *p* = 0.00808; Figure 8C). Further correlation analyses confirmed that the risk score was significantly and positively correlated with TMB (Spearman *p* = 0.00202; Figure 8D).

We further explored the copy number burdens of distinct subgroups. Similar to the findings as previous described, the high-risk BC patients, occupied with non-inflamed TME, showed a higher burden of gain and loss both at the focal and arm-levels (Figure 8E). As illustrated within focal amplification and deletion peaks, we observed the deletions of chr1 (1p36) and amplifications of chr8 (8p11), chr17 (17q12), and chr20 (20q13) were significantly enriched in high-risk patients (Figure 8F, Supplementary Figure S12).

### The Risk Score Distinguished Different Therapeutics Responses in BC

**FIGURE 9:**
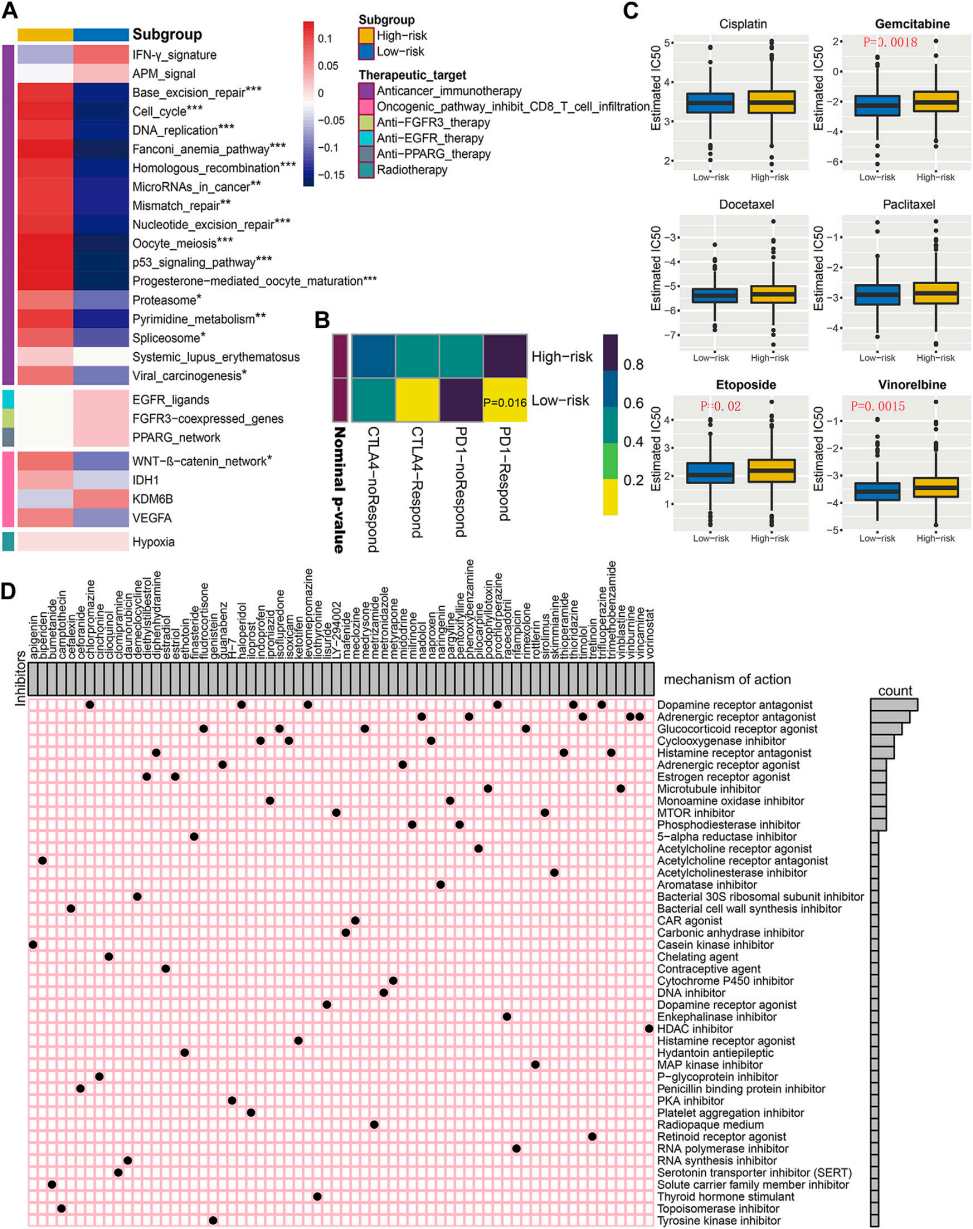
Correlation of established risk score with therapeutic response to several therapies in BC patients. **(A)** Differences in enrichment scores (average z-scores) of several therapeutic signatures for low- and high-risk patients. **(B)** Immunotherapeutic response prediction for anti-PD-1 and anti-CTLA-4 treatments in low- and high-risk patients. **(C)** Differential chemotherapeutic responses for distinct risk subgroups. Boxplots showing the estimated IC50 of risk subgroups for particular chemotherapeutic drugs, including cisplatin, gemcitabine, docetaxel, paclitaxel, etoposide, and vinorelbine. **(D)** Potential molecular inhibitors derived from Connectivity Map analysis (CMap). Based on the differential expression genes between distinct risk subgroups, we utilized the heatmap to depict each compound (perturbagen) from CMap database that shares similar mechanism of action (MoA). Sorted by descending number of inhibitors with shared MoA.

After comparing the differences in enrichment scores of therapeutic signatures, we could determine the roles of risk score in predicting clinical response to these therapies. As exhibited in Figure 9A, the majority of immune inhibited oncogenic pathways, such as WNT-β-catenin_network, cell cycle, mismatch repair, and p53 signaling pathway, were invigorated in the high-risk subgroup, suggesting an inhibited immune status in them. And it may be reasonable to take these signatures as potential therapeutic targets for high-risk BC patients. However, for low-risk ones, immunotherapy should be regarded as candidates due to the inflamed TME within them. The submap analysis also revealed the low-risk patients were more sensitive to anti-PD-1 treatment (Figure 9B).

Since chemotherapy is a commonly used strategy for clinical treatment on BC patients, we selected three commonly used and three other useful chemicals agents (cisplatin, gemcitabine, docetaxel, paclitaxel, etoposide, and vinorelbine), further evaluating the clinical responses of each risk subgroup. As is shown in Figure 9C, the estimated IC50 for gemcitabine, etoposide, and vinorelbine was significantly higher in high-risk BC subgroup, indicating potential chemotherapeutic drug resistance for these patients.

Additionally, we intended to screen for the candidate compounds, especially targeting the high-risk BC patients identified by our risk score. Top 300 DEGs were obtained by comparing the corresponding mRNA expression profiles between low- and high-risk subgroups. The top 66 perturbagens that potentially target these DEGs were exhibited in Figure 9D, along with 48 MoAs derived from CMap analysis. The top hits revealed that six compounds (chlorpromazine, haloperidol, levomepromazine, prochlorperazine, thioridazine, and trifluoperazine) shared the MoA of dopamine receptor antagonist. Meanwhile, other specific MoAs, such as estrogen receptor agonist (diethylstilbestrol and estriol), mTOR inhibitor (LY-294002 and sirolimus), PKA inhibitor (H-7), and tyrosine kinase inhibitor (genistein), were also identified as potential therapeutic targets for high-risk BC patients. These findings may provide opportunities for optimizing treatment in BC.

## Discussion

As a highly complex and heterogeneous disease, BC involves intricate interwoven relationships (Reis-Filho and Pusztai, 2011). Currently, the conventional clinicopathologic indicators, including tumor size, lymph node metastasis, TNM stage, and cellular biomarkers (ER, PR, HER-2 status and Ki-67 index) of tumor biopsy, are still the gold standard for the risk stratification and subsequent formulating of therapeutic scheme. However, BC patients might possess different treatment responses and clinical outcomes spanning <6 months to beyond 10 years, even with the same histological grade and pathological stage (Network, 2012; Olsson et al., 2013). In this aspect, integrating reliable prognostic biomarkers into real-life work are a critical requisite to identifying the subset of patients who harbored a worse prognosis and might benefit from the systematic adjuvant therapy. Significant research on the high-throughput gene expression profiles has led to recent commercialization of two multi-gene-based signatures (Mamounas et al., 2010; Cardoso et al., 2016; Geyer et al., 2018), but their additional utility in aiding treatment decision in BC patients still needs validating based on long-term prospective studies (Sparano et al., 2015).

DNA methylation, as a changeable and possibly heritable epigenetic modifying mechanism, offers promising clues for early detection, monitoring treatment response, prognosis prediction, and molecular subtyping in cancer (Franco et al., 2008; Wu and Ni, 2015; Zhang et al., 2018c). The aberrant methylation of particular subsets of CpG sites is prone to take place at the beginning of carcinogenesis, resulting in specific processes of tumorigenesis (Wang et al., 2019). Preliminary investigation indicated that using DNA methylation as a biomarker holds serious implications for cancer diagnosis and treatment, including the relative steady expression both *in vivo* and *ex vivo* (Keeley et al., 2013), ensuring precise judgement in a minimally invasive way instead of requiring bulk tissue samples (Dai et al., 2011), and higher accuracy in cancer management, especially distinguishing relatively indolent or aggressive tumors (Hao et al., 2017). Taking the potential epigenetic modification of DNA methylation in breast carcinogenesis into account, it is reasonable to consider the CpG sites to increase the effectiveness of adjuvant therapy. In the current study, we proposed a robust, individualized prognostic signature that can estimate OS probability in BC patients on the basis of 10 aberrant CpG sites, through integrating the well-established public, large-scale multi-omics sequencing cohorts. And our prognostic signature could further stratify clinically defined groups of patients (e.g., age, histological type, TNM stage) into subgroups with distinct survival outcomes. Moreover, attributed to the complementary value of molecular and conventional clinical characteristics, our inclusive nomogram could indicate a more accurate estimation of OS in both short-term and long-term prognosis prediction for BC patients.

We applied a three-stage selection procedure to screen out the prominent biomarkers from more than 300,000 CpG sites after quality control and rigorous filtering. Initially, in the discovery stage, differential methylation analyses using paired tissue profiles winnowed out nearly 99.86% of probes, and those CpG sites with cancer-specific properties in BC patients were preserved. Next, in the training stage, univariate Cox regression analyses were performed to assess their prognostic values, excluding probes unrelated to OS. Meanwhile, considering that the Cox model is insufficient for variable selection due to the high-dimensional data, the successive application of machine-learning methods, LASSO-penalized Cox regression with 10-fold cross validation, to screen the optimal combination of CpG sites for further modeling, markedly raised the accuracy of methylation-based signature. Finally, in the validation stage, the performance of the established model was further validated based on two external independent cohorts, confirming its robust and reliable prognosis prediction.

Furthermore, the GSEA analysis provided more opportunities for deciphering the largely untapped mechanisms which our identified CpG sites might participate in. For instance, Nothch4 signaling pathway is considered as playing pivotal roles in malignant potential, including proliferation, invasiveness, and metastasis, by sustaining epithelial-mesenchymal transition and controlling BC stem cell activity (Nagamatsu et al., 2014; Kim and Singh, 2015; Wang et al., 2018; Giuli et al., 2019), and activation of it could contribute to endocrine therapy assistance in BC cells (Lombardo et al., 2014; Simões et al., 2015; Bui et al., 2017). Upregulation of ORC1 (origin recognition complex 1), the largest unit of ORC required in the initiation of DNA replication, was gradually confirmed resulting in DNA re-replication to trigger DNA damage response and control cancer cell-cycle (McNairn and Gilbert, 2005; Kara et al., 2015; Chen et al., 2019). Altered tumor metabolism, including increased glycolysis in cancer cells, determined the malignant biological behaviors and cancerous phenotype (Ma and Zong, 2020). Inhibitor of glycolysis pathway could sensitize the anti-cancer effect of chemotherapeutic agents and delay the occurrence of acquired drug resistant in hypoxia (Le Calvé et al., 2010; Jiang et al., 2018). In addition, dysregulation of other pathways, such as regulation of apoptosis (Jordan, 2015; Ahmed et al., 2019), DNA damage checkpoints (Lord and Ashworth, 2012; del Rincón et al., 2014), and P53 signaling pathway (Silwal-Pandit et al., 2017), were also involved in cancer cell growth, proliferation, invasion, and metastasis and played crucial roles in BC cells. Hence, the results mentioned above added more evidence for the interactions between our established signature and cancers, highlighting its clinically transitional potential.

Nowadays, exploiting the intrinsic interplay of host immune system and malignant tumors has achieved impressive success (Chung et al., 2017; Plava et al., 2019). Moreover, preliminary reports have provided elegant analyses on the cross-talk between tumor-intrinsic genes and BC microenvironment (Fox et al., 2019; Patel et al., 2019), hailing cancer immunotherapy as a vast breakthrough in the combat against malignancies (Jia et al., 2017; Szekely et al., 2018). However, currently, the immune checkpoint blockades such as PD-1, PD-L1, and CTLA-4 do not exhibit an overwhelming situation for conquering cancers. Only a small portion of BC patients with suitable immunogenicity could show clinical response to immunotherapy. And it is necessary for the success of immunotherapy that to hold an inflamed TME in conjunction with pre-existing anticancer immunity. Particular molecules or pathways, inducing the formation of non-inflamed TME and making the recruitment of tumor-cytotoxic T cells invalid, may cause resistance to immunotherapeutic agents. Intriguingly, an extensive immunogenomic analysis has revealed that our established risk score could distinguish subgroups with different prognoses and remarkably distinct phenotypes within TME. For high-risk BC patients with a non-inflamed TME, transforming it into an inflamed TME is one of the top priorities along with re-invigoration of tumor-infiltrating immune cells to drive tumor regression. To some extent, these findings suggested the CpG sites included in our signature hold great promise for identifying novel molecular targets and improving cancer management in the era of immunotherapy.

Although our study proposed a robust 10*-*CpG-based prognostic signature and shed new light on the epigenetic microenvironment for possible therapeutic potential, several limitations should be addressed. Firstly, due to the retrospective nature of our work, it is imperative to design multicenter prospective clinical trials with large sample sizes for further providing high-level evidence for clinical application. Secondly, due to the lack of normal mammary samples, the differential methylation profiles merely derived from the comparison between tumor and adjacent normal tissues need to be further validated. Thirdly, our attempts were based on single-omics data (DNA methylation), so that the understanding of BC-related properties could be inevitably incomprehensive in the presence of tumor heterogeneity. Finally, concrete biological mechanisms of the candidate CpG sites still need to be experimentally verified, especially underlying the immune microenvironment.

## Conclusion

Overall, we constructed and externally validated a novel predictive model by sufficiently integrating and analyzing the DNA methylation profiles. And we expect that application of our model will not only greatly contribute to the personalized follow-up and decision-making process of clinicians, but also facilitate further understanding of the basic biology of BC and thereby inform the drafting of appropriate therapeutic strategies in the future.

## Data Availability

Publicly available datasets were analyzed in this study. This data can be found here: The data sets involved in our study are publicly available in the TCGA database (https://cancergenome.nih.gov/) and the GEO database (https://www.ncbi.nlm.nih.gov/geo/).

## Abbreviations

AUC, area under curve; BC, breast cancer; CI, confidence interval; c-index, concordance index; CpG, 5′-cytosine-phosphate-guanine-3′; DCA, decision curve analysis; ER, estrogen receptor; ESTIMATE, estimation of stromal and immune cells in malignant tumor tissues using expression data; FDR, false discovery rate; FISH, fluorescence *in situ* hybridization; GEO, gene expression omnibus; GSEA, gene set enrichment analysis; KNN, k-nearest neighbors; Her-2, human epidermal growth factor receptor-2; HR, hazard ratio; IHC, immunohistochemical; LASSO, least absolute shrinkage and selection operator; MCP-counter, microenvironment cell populations-counter; MSigDB, molecular signatures database; NES, normalized enrichment score; OS, overall survival; PR, progesterone receptor; ROC, receiver operator characteristic; SNP, single-nucleotide polymorphisms; TCGA, the cancer genome atlas; TPM, transcripts per kilobase million.
