# Supplementary material for: A High Epigenetic Risk Score Shapes the Non-Inflamed Tumor Microenvironment in Breast Cancer
Source: Front Mol Biosci. 2021 Jul 26;8:675198. doi: 10.3389/fmolb.2021.675198 (PMC8350480; doi:10.3389/fmolb.2021.675198)
Supplement: Supplementary file 1 [file DataSheet1.docx]

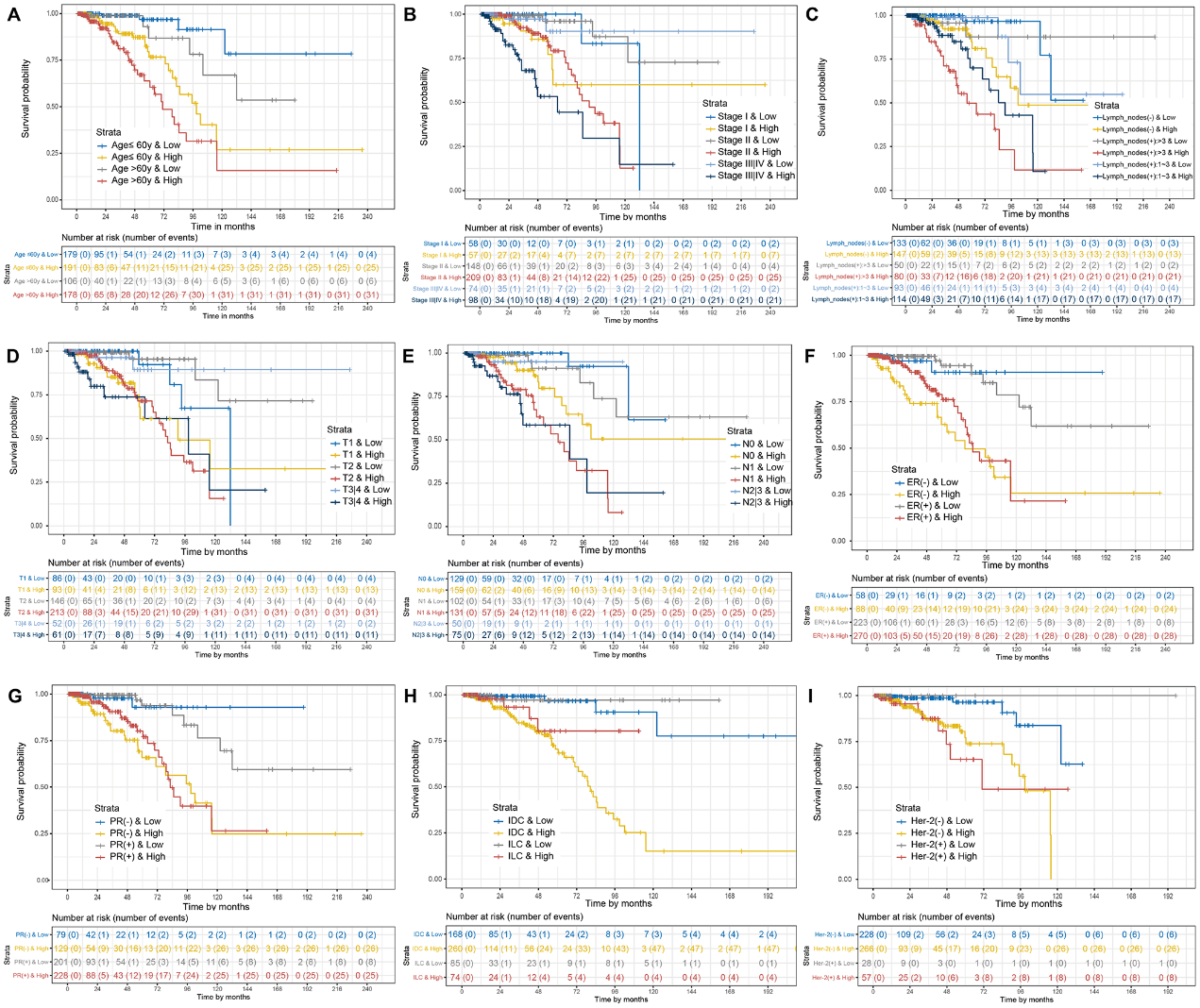


**Supplementary Figure S1**. Subgroup and stratification analyses of 10-CpG-based signature. Kaplan-Meier curves plotting overall survival for BC patients according to the 10-CpG-based signature in subgroups stratified by (A) age, (B) pathologic stage, (C) regional lymph nodes involvement, (D) T stage, (E) N stage, (F) ER status, (G) PR status, (H) histological type and (I) Her-2 status.


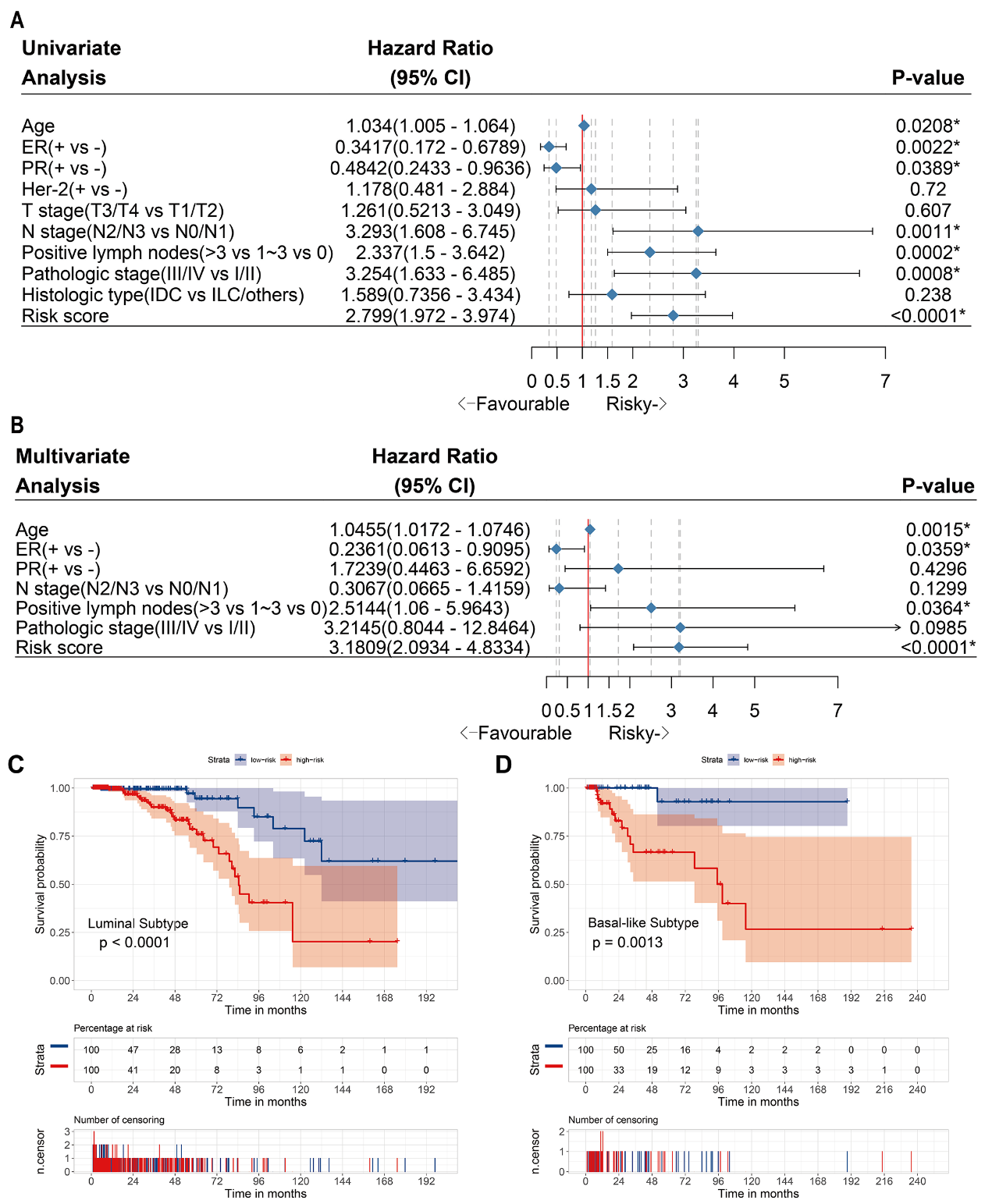


**Supplementary Figure S2.** The 10-CpG-based signature is independent of clinicopathological features for predicting prognosis. The univariate followed by multivariate Cox regression analysis of clinicopathological characteristics and 10-CpG-based signature in the TCGA cohort. (A) Forest plot depicting the results of univariate Cox analysis of available clinicopathologic features and 10-CpG-based signature on overall survival. (B) Multivariate regression analysis of the relation between 10-CpG-based signature and clinical risk factors regarding prognostic value. The boxes and width of the horizontal lines represent the hazard ratio (HR) and 95% confidence interval (CI), respectively. The red vertical line indicates the HR of 1.0. (C) Stratified Cox analysis of prognostic signature in luminal A and B subtype. (D) Stratified Cox analysis of prognostic signature in basal-like subtype.


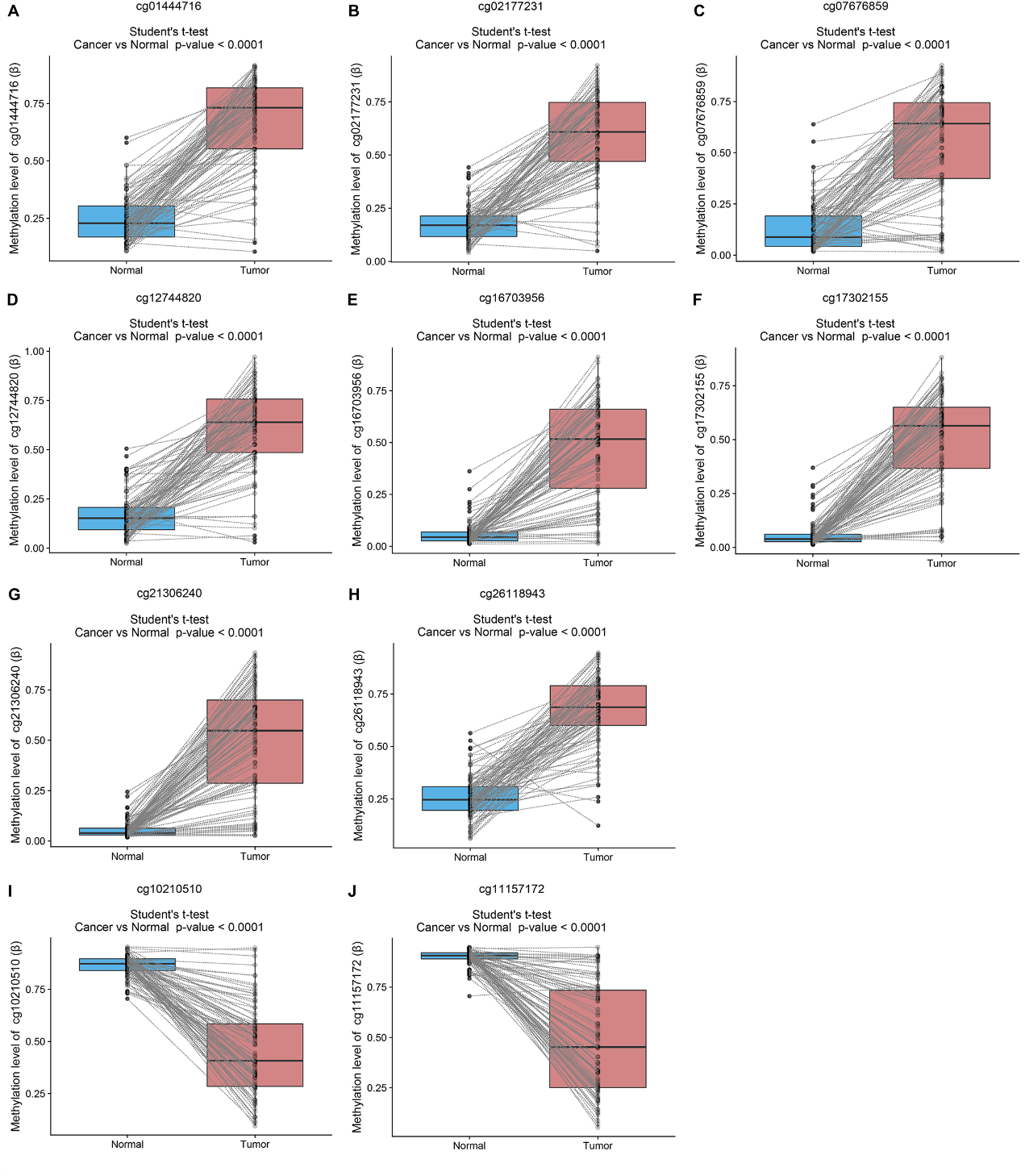


**Supplementary Figure S3.** The difference of methylation level (β) of CpG sites between primary BC and corresponding adjacent normal tissues, which were all identified as candidates for constructing prognostic signature in the training cohort. Paired Student’s t-test was used for data analysis.

**
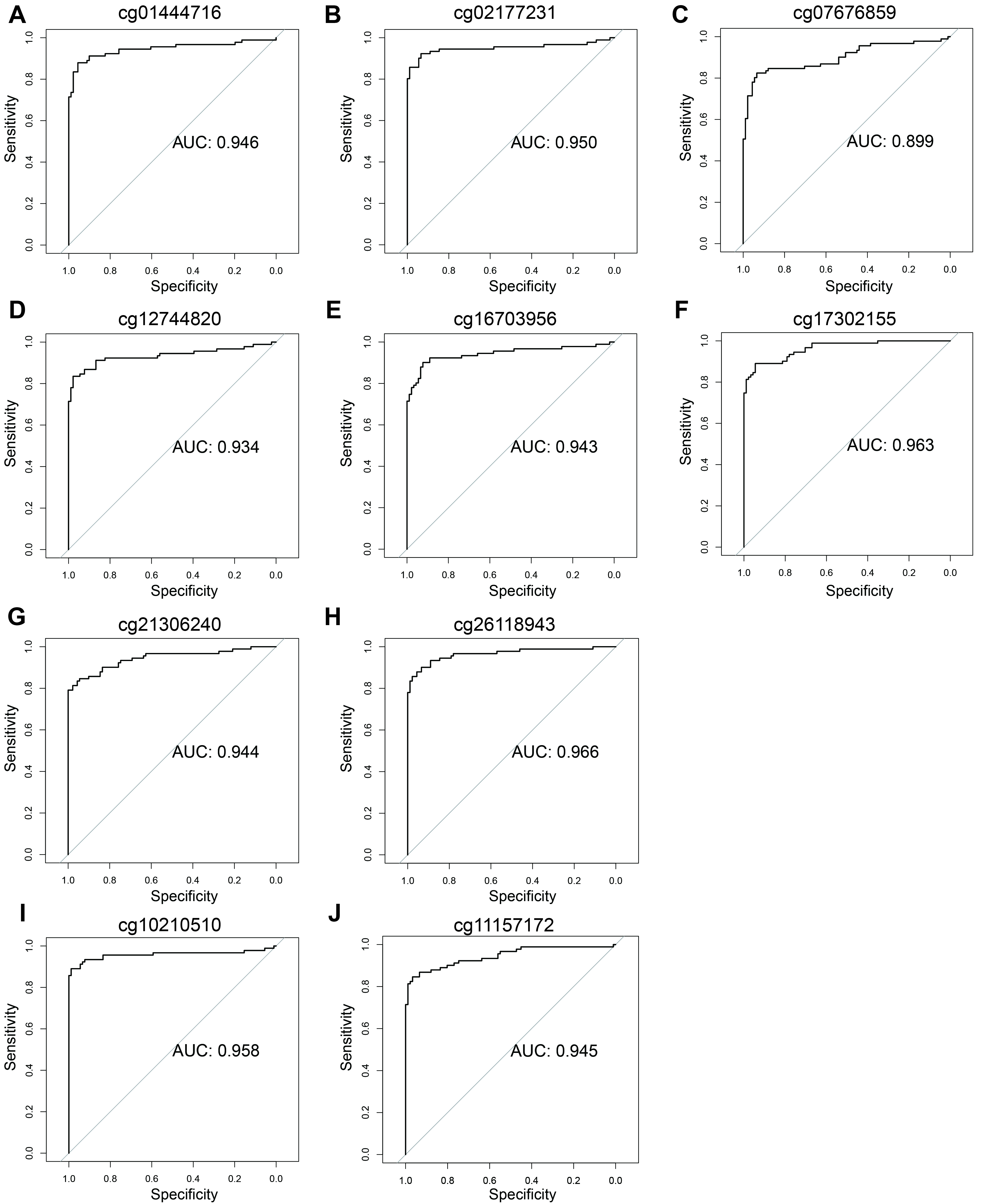
**

**Supplementary Figure S4.** (A-J) ROC curve of candidate CpG sites to depict predictive efficiency upon diagnosis of BC in discovery set, respectively.


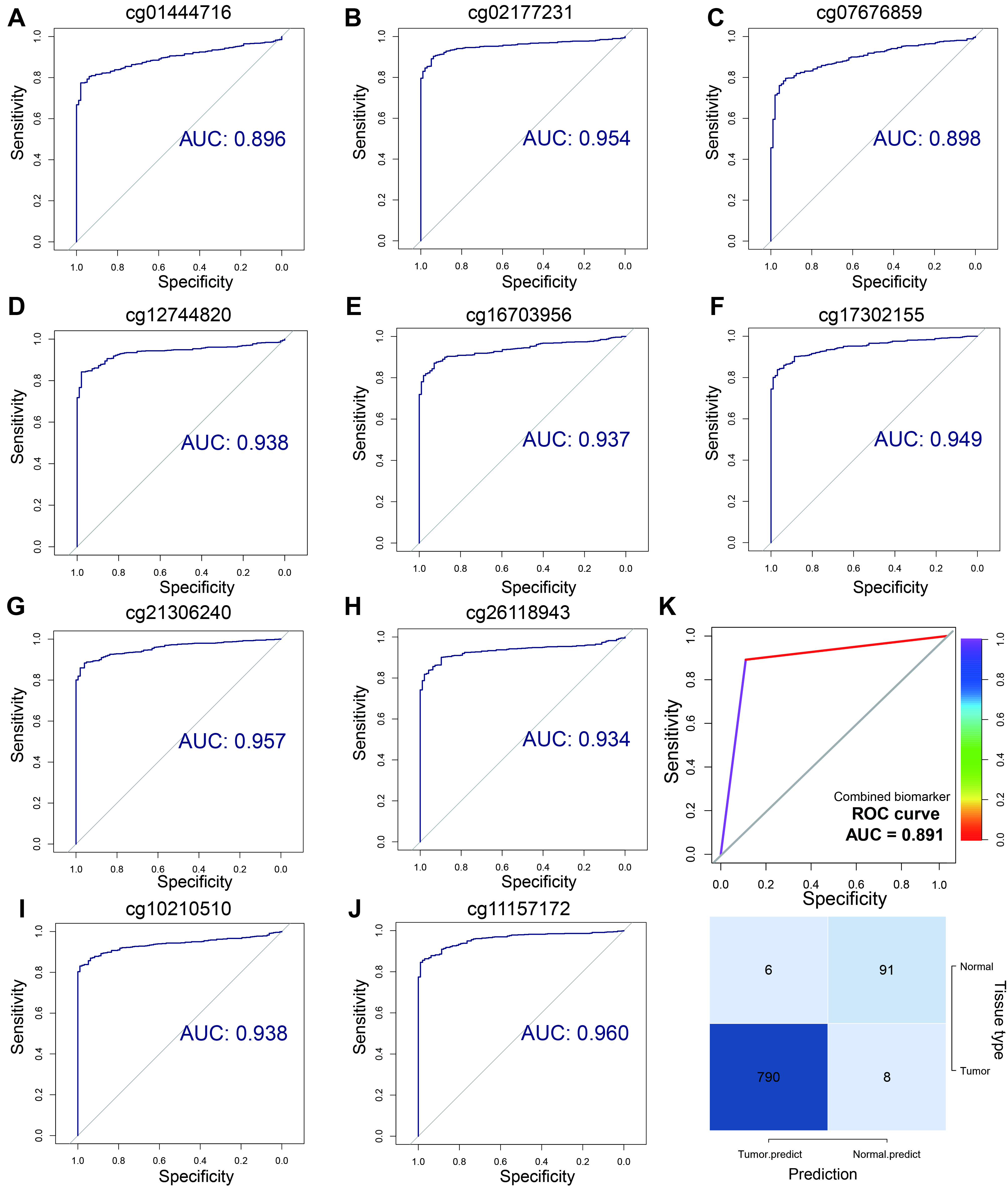


**Supplementary Figure S5.** (A-J) ROC curve of candidate CpG sites with AUC value calculated to validate predictive efficiency upon diagnosis of BC in entire TCGA cohort, respectively. (K) ROC curve of combined 10-CpG-site biomarker in diagnosing breast cancer in entire TCGA cohort, with contingency table estimating the consistency between predicted sample type of combined 10-CpG-site biomarker and actual tissue type.


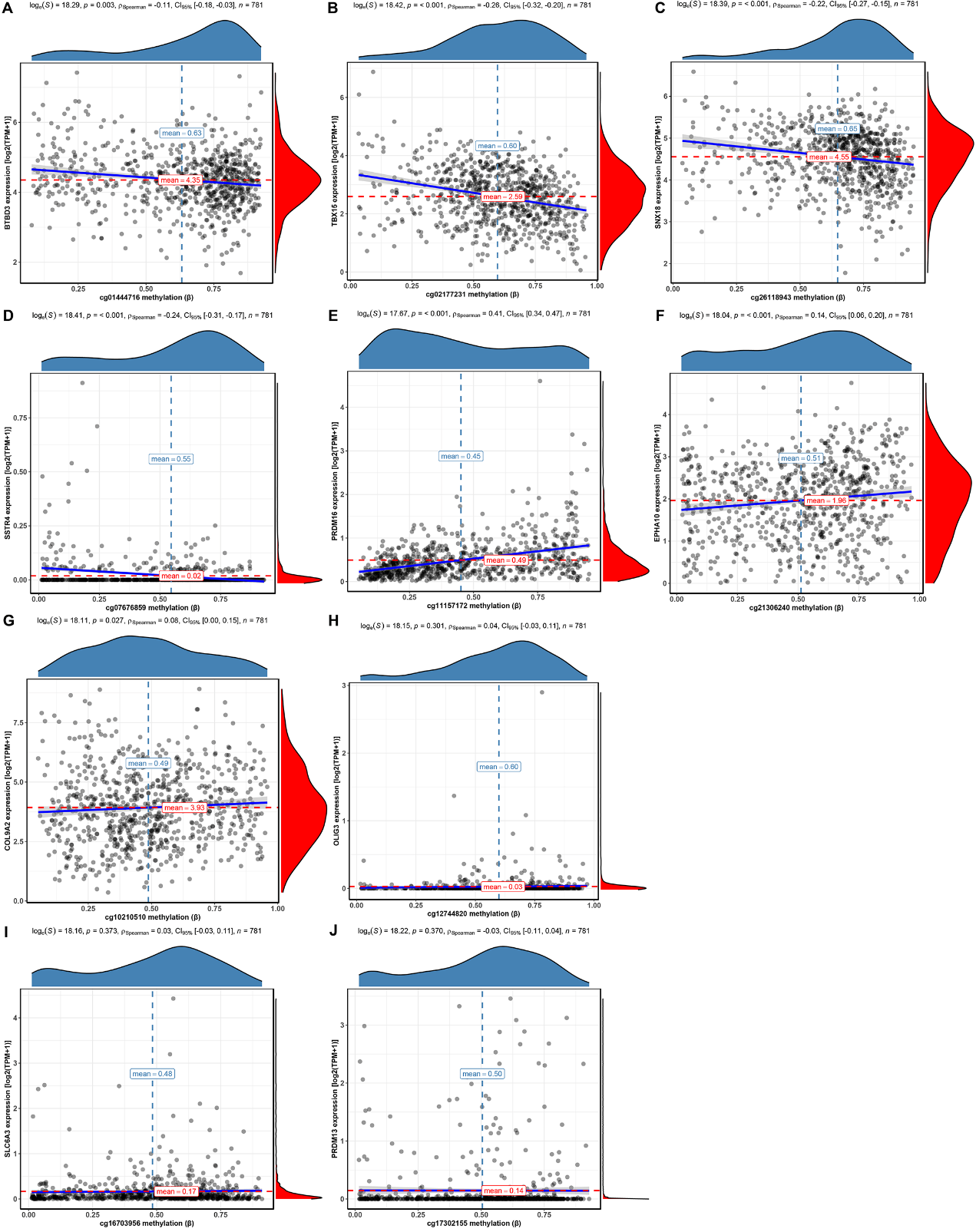


**Supplementary Figure S6.** The correlation analyses between the methylation level (β) of particular CpG sites involved in 10-CpG-based prognostic signature and the normalized expression level (log2(TPM+1)) of parent genes. Correlation coefficients and corresponding p-values were computed via Spearman and distance correlation analyses.


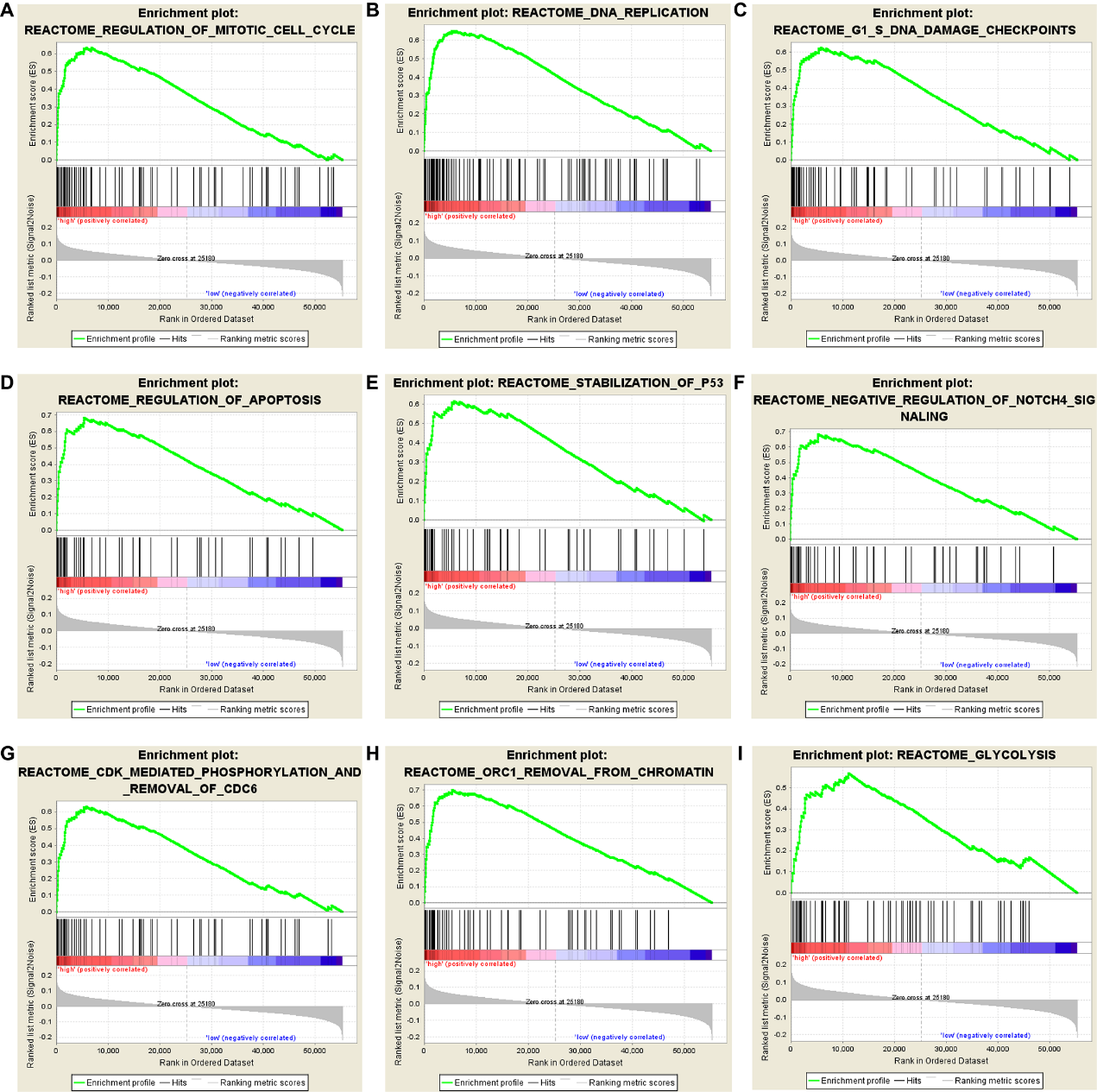


**Supplementary Figure S7.** Enrichment plots depicting the significantly enrichment results which are positively correlated with *10-*CpG-based signature.


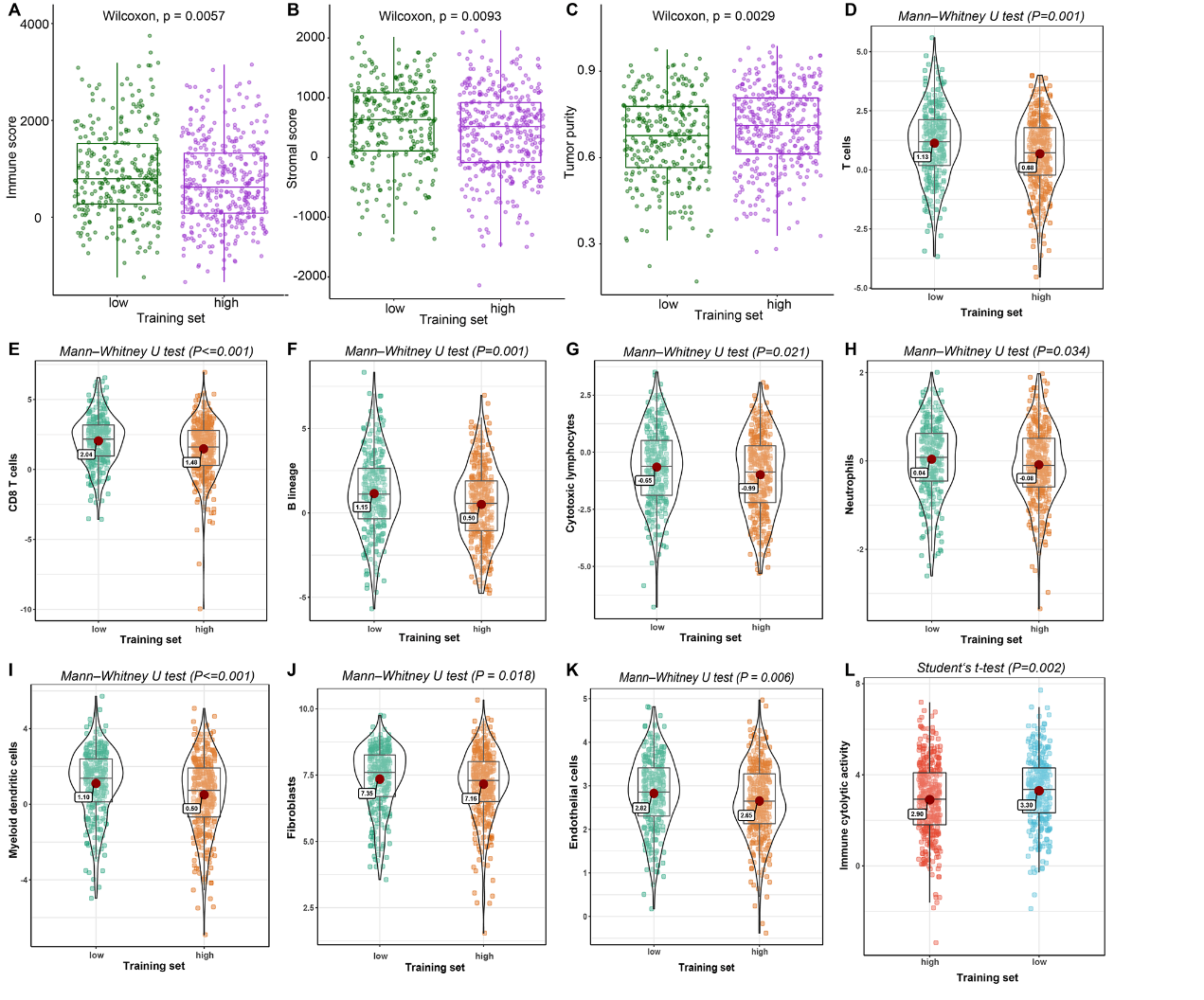


**Supplementary Figure S8.** The estimation of immune infiltration in high- and low-risk BC patients. (A-C) The inferred immune score, stromal score and tumor purity between low- and high-risk patients, respectively. Wilcoxon rank-sum test was used to estimate the statistical significance of difference. (D-K) Violin plot visualizing the significantly different immune and stromal cells, which were inferred by MCP-counter, between low- and high-risk subgroup, including (D) T cells, (E) CD8+ T cells, (F) B cell lineage, (G) cytotoxic lymphocytes, (H) neutrophils, (I) myeloid dendritic cells, (J) fibroblasts and (K) endothelial cells. Comparisons between different subgroups were performed with Mann-Whitney U test. (L) Box plots depicting the association between immune cytolytic activity and subgroups classified by *10-*CpG-based signature. Comparisons between subgroups were performed by Student’s t-test.


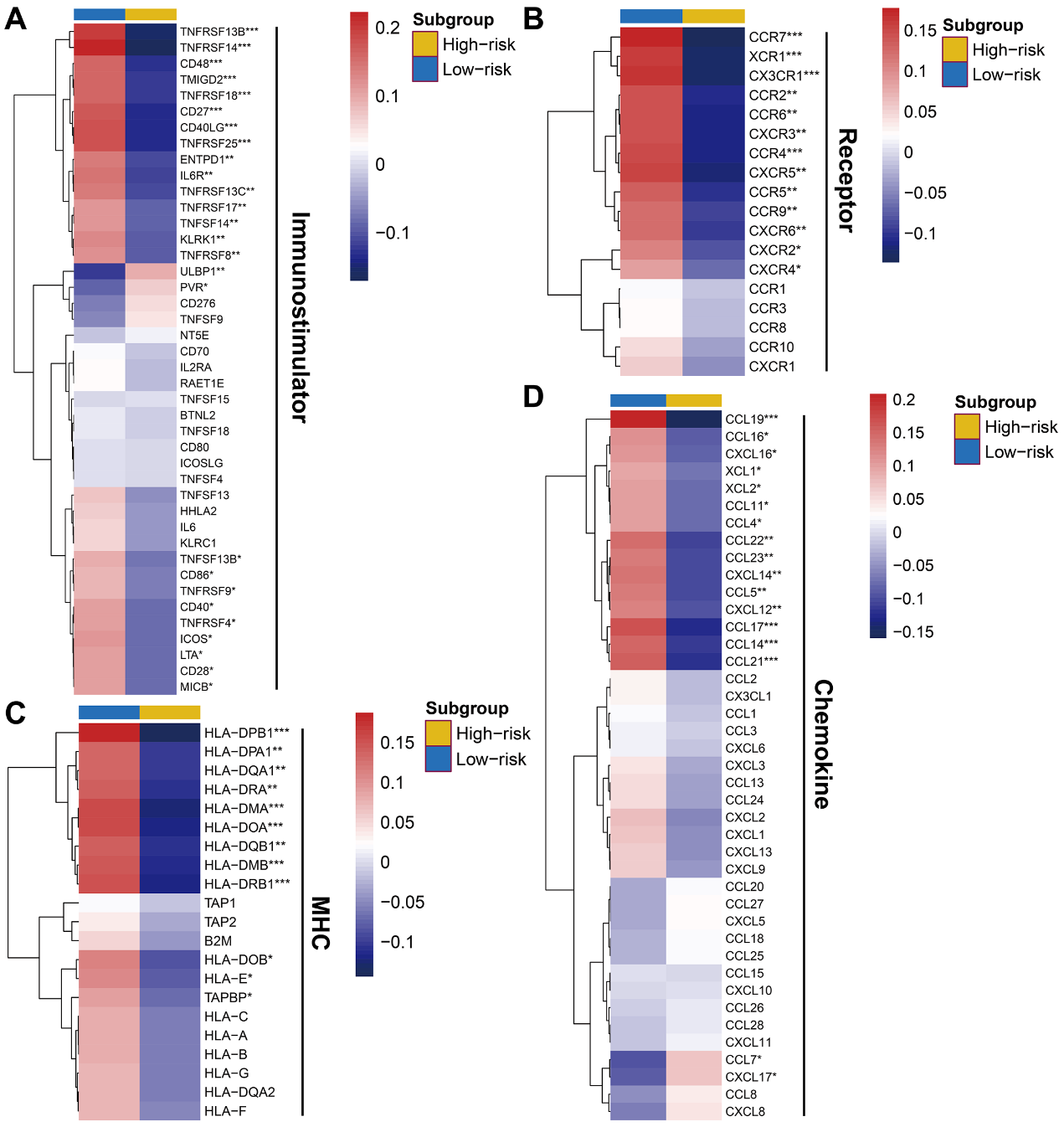


**Supplementary Figure S9.** Differences in the expression (average z-score) of 122 immune-related modulators between low- and high-risk BC patients, including immunostimulators (A), receptors (B), MHC molecules (C), and chemokines (D). The asterisks indicated the statistical significance of pairwise correlations or comparisons (*P < 0.05; **P < 0.01; ***P < 0.001).


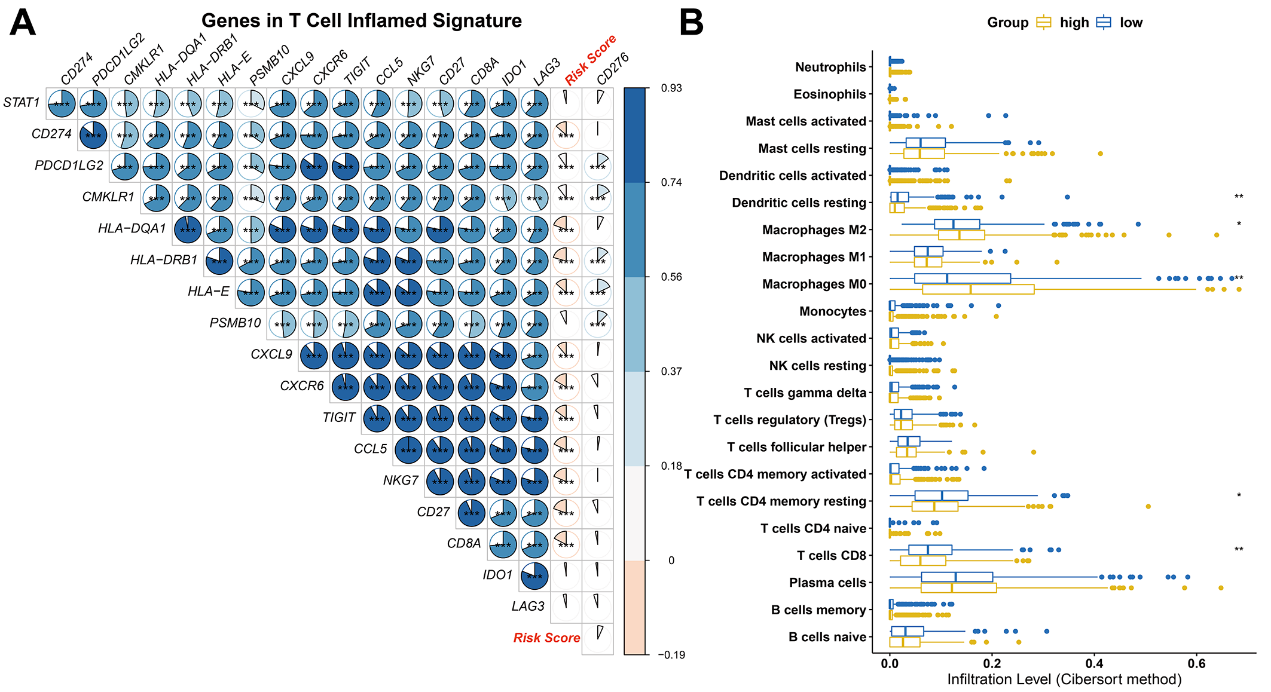


**Supplementary Figure S10.** (A) Correlation between expression levels of individual genes included in pan-cancer T cell inflamed signature and risk score in BC patients. (B) Differences in the infiltration levels of 22 immune cells (quantified by Cibersort algorithm) between low- and high-risk subgroups. The asterisks indicated the statistical significance of pairwise correlations or comparisons (*P < 0.05; **P < 0.01; ***P < 0.001).


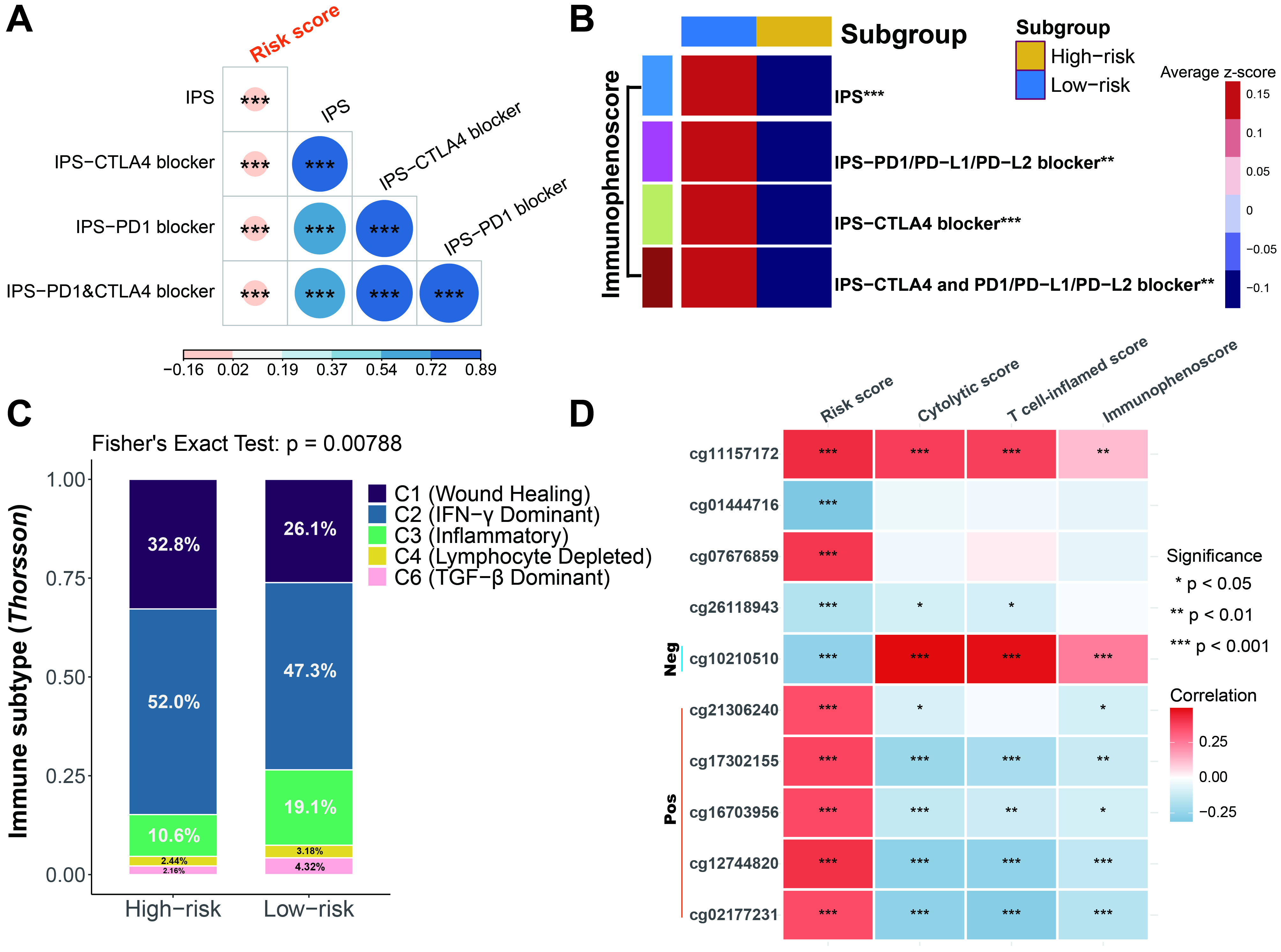


**Supplementary Figure S11.** (A) Correlation between risk score and categories of immunophenoscore, including IPS, IPS-CTLA4 blocker, IPS-PD1-PD-L1-PD-L2 blocker, and IPS-PD1-PD-L1-PD-L2-CTLA4 blocker scores. (B) Comparisons of IPS, IPS-CTLA4 blocker, IPS-PD1-PD-L1-PD-L2 blocker, and IPS-PD1-PD-L1-PD-L2-CTLA4 blocker scores between low- and high-risk BC patients. (C) The comparison of distribution of immune subtype between low- and high-risk subgroups. (D) Heatmap shows the positive (red) and negative (blue) correlation between methylation levels of each CpG site within final signature and adverse indicators of non-inflamed TME, including immune cytolytic score, T cell-inflamed score and immunophenoscore. * means P < 0.05, ** means P < 0.01, and *** means P < 0.001.


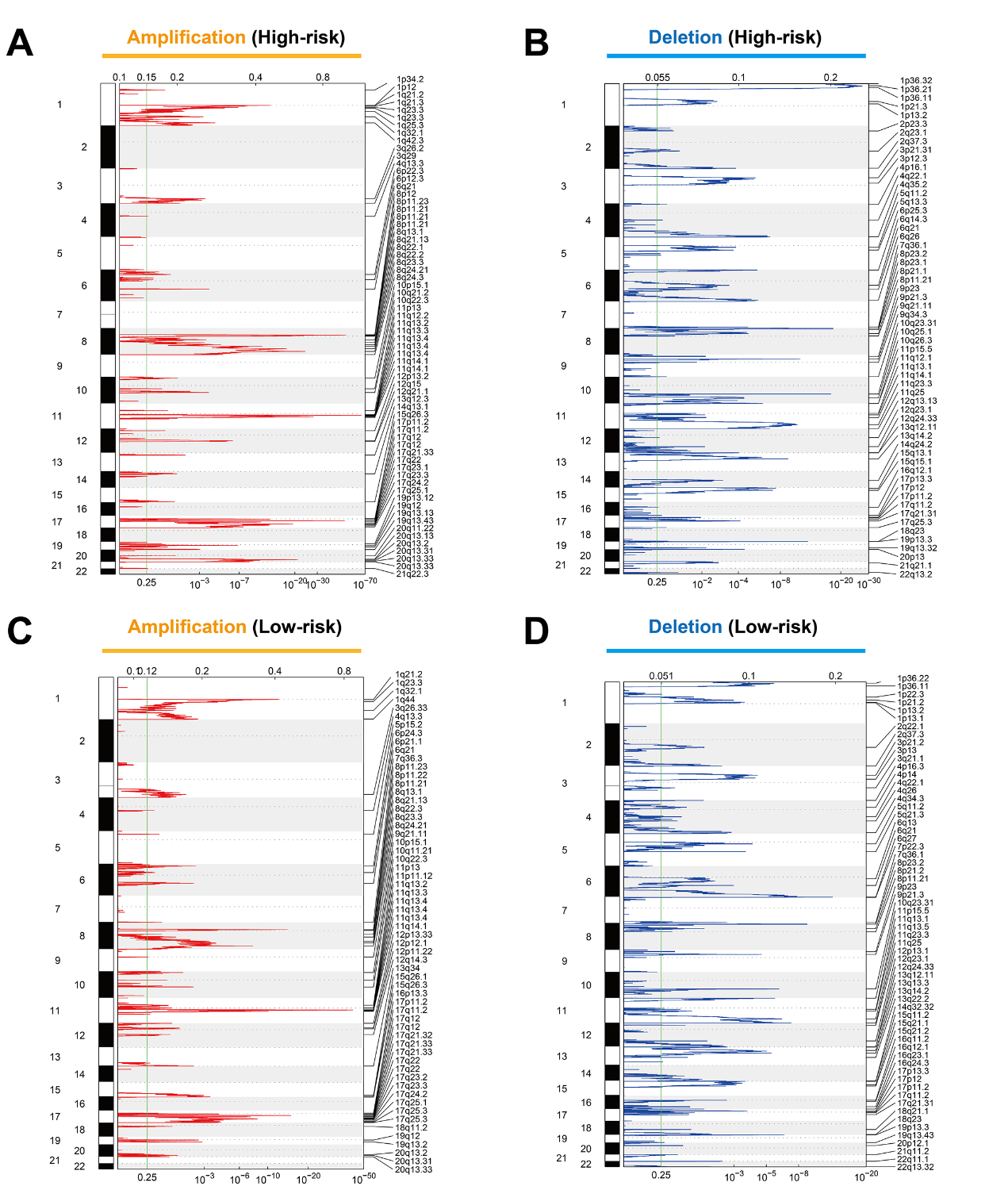


**Supplementary Figure S12.** Significant broad and focal copy number alterations in genome of distinct prognostic subgroups. Detailed amplifications (red) and deletions (blue), generated with GISTIC_2.0 software, are displayed across the genome (chromosomal position, indicated by the y-axis) for BC patients. The q-value of each locus for cytobands is indicated by x-axis.
